# Evaluation of Orientation-Dependent Cation−π Pairwise Effects within Collagen Triple Helices

**DOI:** 10.1021/acs.jpcb.4c08691

**Published:** 2025-04-30

**Authors:** Tzu-Jou Yao, Yung-En Ke, Wen-Ling Lin, You-Cheng Lin, Chih-Han Yang, Tsai-Ling Hsu, Jia-Cherng Horng

**Affiliations:** † Department of Chemistry, National Tsing Hua University, Hsinchu 300044, Taiwan R.O.C; ‡ Frontier Research Center on Fundamental and Applied Sciences of Matters, National Tsing Hua University, Hsinchu 300044, Taiwan R.O.C

## Abstract

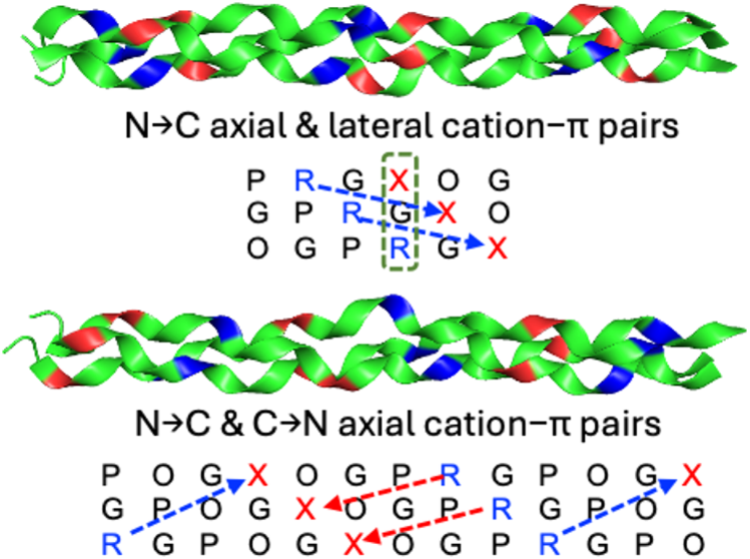

Various noncovalent interactions have been introduced to explore their impacts in folding a collagen triple helix. Among these interactions, the cation−π interaction represents one of the compelling forces stabilizing the triple helix. Still, the effects depend on the pairwise components and the orientation between the cationic and aromatic moieties. To gain more insights into this interaction within a collagen trimer, we prepared a series of collagen-mimetic peptides (CMPs) with cationic residues and aromatic residues incorporated to examine the contributions of two types of axial cation−π pairs (N → C and C → N cationic-to-aromatic pairwise) and the lateral cation−π pair. Circular dichroism (CD) measurements indicate that the N → C axial pairs have a significant stabilization effect. In contrast, the lateral and the C → N axial pairs destabilize the fold, and the lateral pairs cause the most destabilization consequences. We further designed and prepared the CMPs containing various lateral and axial cation−π pairs to investigate the coupling consequences in homotrimers and heterotrimers. From CD data, we found that the predicted differences in melting temperatures using individual cation−π pairwise contributions were comparable to the observed values for the designed homotrimers. CD and NMR measurements showed favorable cation−π interactions could effectively induce the folding of heterotrimers, in which the CMPs with more N → C axial pairs formed a more stable trimer than those containing a smaller number of N → C axial pairs. In this study, we have disclosed more valuable information about the properties of cation−π pairwise effects within a collagen triple helix, which can be considered in designing collagen-related peptides and materials.

## Introduction

Collagen, a key component of the extracellular matrix, represents the most abundant protein in mammals, and 28 types of collagen have been identified.^1^ The right-handed triple helix consists of three left-handed polyproline II helices.^2^ Possessing high biocompatibility for potential biomedical applications, collagen is an attractive target for study; in particular, collagen-mimetic peptides (CMPs) have been widely used as models for studying collagen-related materials.^3−6^ Recently, a few studies have focused on using covalent and noncovalent interactions to stabilize collagen trimers,^7−10^ providing new valuable information for the design of collagen-related materials. In addition to homotrimers, heterotrimers are predominant in collagen. Thus, various forces have also been applied to induce the CMPs to form the folds that mimic collagen heterotrimers, including electrostatic interactions,^11−18^ covalent capture,^19,20^ and cation−π interactions.^21,22^ Among the forces is the cation−π interaction, which was first shown to stabilize homotrimers by our laboratory.^23^ We further utilized this interaction to promote the trimer assembly^24^ and induce the formation of heterotrimers.^21,22^ A later study by Xu et al. used collagen heterotrimers to screen for optimum cation−π interactions to stabilize the triple helix.^25^ It is clear from the above review that the cation−π interactions within collagen triple helices have received much attention.

**Figure 1:**
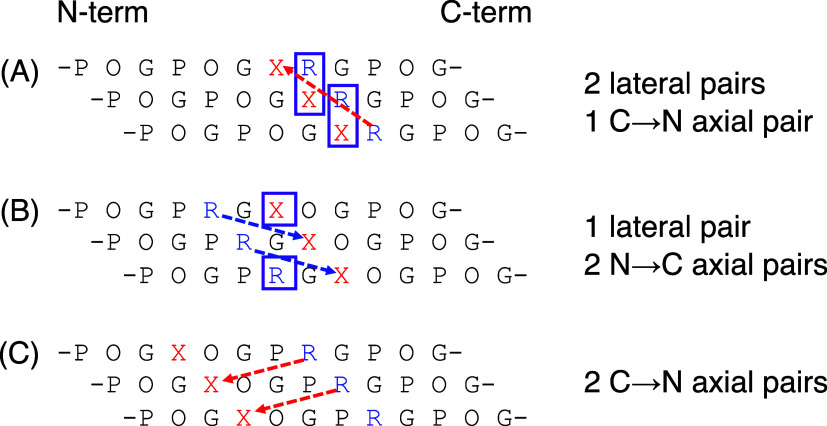
Illustration of cation−π pairs with different orientations within a collagen triple helix. O is (2*S*,4*R*)-hydroxyproline and X is an aromatic residue. The boxes indicate the lateral pairs, the blue arrows indicate the N → C axial pairs, and the red arrows indicate the C → N axial pairs.

Aromatic residues are relatively rare in collagen; for instance, Phe and Tyr only account for 1.4 and 0.3% of total residues in the collagen inside mammalian bone,^26^ but they appear to play a critical role in stabilizing collagen structures and assembly.^27−29^ In contrast, Arg residues occur quite frequently in collagen,^30^ suggesting that favorable cation−π interactions could form or be introduced if the Arg-aromatic pairs were properly arranged in the collagen strands. A recent work by Hartgerink et al. revealed that the cation−π pairs installed in a collagen triple helix can form two pairwise interaction orientations, lateral and axial,^31^ as shown in Figure 1B. The axial pair is defined as the interaction between *i* and *i* + 3 residues according to a typical polyproline II helix.^32^ These studies indicate that the axial pairwise interaction has a stabilization effect on the trimer, but the lateral pairwise interaction has a destabilization effect on the trimer. In a recent study, they further combined the axial cation−π interactions and electrostatic interactions to design heterotrimers.^33^ The exciting results provide new insights into this noncovalent interaction within collagen. In their study, however, only the lateral and the N-terminus cationic to C-terminus aromatic (N → C) axial pairwise interactions (Figure 1A,B) were examined. According to the arrangements of three collagen strands, the C-terminus cationic to N-terminus aromatic pair represents another potential axial (C → N) arrangement, as shown in Figure 1A,C; this pair may contribute to the triple helix stability. We examined the sequences of α chains in type I, II, III, and IV collagens and found the combined occurrence frequencies of GFR and GYR were 6, while those of RGF and RGY were 23, respectively (as shown in Table S1 of the Supporting Information). These sequences are like those illustrated in Figure 1A,B. In addition, the sequence FX′GY′RG, like that in Figure 1C, was also found 9 times in these collagen chains. This finding implied that various pairwise cation−π arrangements might play different roles in collagen. Therefore, it was worthwhile to perform a study to develop insights into this pairwise impact and give a more detailed picture of the effects of incorporating various cation−π pairs into a collagen triple helix.

To explore how the C → N axial pair affects the collagen triple helix, we prepared a series of CMPs containing arginine or aromatic residues to investigate their contributions as well as the N → C axial and lateral pairs. After determining the impact of each cation−π pair, we also prepared CMPs containing Arg and aromatic residues to form homotrimers and then used the contribution of each pair to compare the thermal stability between the homotrimers containing various pairwise cation−π arrangements. Additionally, we also combined the contributions of Arg-Tyr pairs with different orientations to examine their effects on cation−π interaction-induced heterotrimers. From such a comprehensive investigation, we produced new insights into the cation−π pairwise effects within collagen triple helices.

## Materials and Methods

### General

Chemical reagents and Fmoc-protected amino acids were purchased from Acros, AgeneMax, Aldrich, Alfa Aesar, Creo Salus, ECHO, Fluka, J.T. Baker, Merck, and Sigma-Aldrich, and used without further purification. ^15^N-isotopically labeled Fmoc-Gly-OH was purchased from AnaSpec Inc.

### Peptide Synthesis and Purification

All peptides were synthesized on a 0.03–0.1 mmol scale by solid phase peptide syntheses, using the Rink amide MBHA resin or NovaSynTGR resin. Ethyl cyanohydroxyiminoacetate (Oxyma)/*N*,*N*-diisopropylcarbodiimide (DIC)-mediated coupling reactions were used in a microwave peptide synthesizer (CEM, Discover SPS), while HATU-mediated coupling reactions were used in an Automated Peptide Synthesizer PS3 (Protein Technologies) and manual syntheses. The N-termini of all the peptides were acetylated with acetic anhydride upon completion of the final coupling reaction. Cleavage of the peptides from resin and removal of side chain protecting groups were performed with either a solution of 94% TFA/1% TIS/2.5% H_2_O/2.5% ethanedithiol (v/v) or a solution of 95% TFA/2.5% TIS/2.5% H_2_O. The use of amide resin generated an amidated C-terminus upon the cleavage. Reverse phase HPLC with Vydac, Dr. Maisch GmbH, or Cosmosil semipreparative C18 columns was used to purify the crude peptides. H_2_O/acetonitrile gradients with 0.1% (v/v) TFA were used as eluent. Molecular weights of the synthesized peptides were confirmed by MALDI-TOF mass spectrometer (Bruker Daltonics, Autoflex III Smartbeam LRF 200-CID). The measured molecular weights are listed in Table S2 of the Supporting Information. According to analytical HPLC analysis, all peptides were more than 90% pure (Figures S1–S6 in the Supporting Information).

### Circular Dichroism (CD) Spectroscopy

CD measurements were performed by an AVIV model 410 CD spectrometer with a 1 mm path length quartz cuvette. Peptides were dissolved in pH 7.0 and 20 mM sodium phosphate buffer to prepare the concentration of 0.2 mM for CD measurements. The pH 7.4 buffer with 20 mM sodium phosphate was used for heterotrimer samples. The samples were heated to 80 °C for 20 min to denature the triple helices, followed by cooling to room temperature for 3 h and then incubation at 4 °C for at least 1 week before CD measurements to allow the complete formation of triple helices. For thermal unfolding experiments, the CD signals at the wavelengths (224 to 226 nm) with the maximum ellipticity of individual CMPs were monitored, and the data were recorded every 1 °C with an average heating rate of 0.16 °C/min. Values of melting temperature (*T*_m_) were determined by two-state model fitting.^10^

### Differential Scanning Calorimetry (DSC) Analysis

DSC thermograms were acquired by VP-DSC (MicroCal) at the National Chung Hsing University Instrumentation Center, and the data were analyzed in affiliated Origin software. Before DSC measurements, sample solutions with peptide concentrations of 0.6 mM were prepared similarly to those for CD measurements. The peptide solutions were degassed for over 5 min before measurements. Samples were scanned from 10 to 80 °C (homotrimer samples) with a heating rate of 0.1 °C/min. A progress baseline was subtracted from each DSC curve before analysis. The *T*_m_ value was obtained at the maximum of each transition in the DSC endotherm. Values of Δ*H* (per mole of monomer) were obtained by directly integrating the DSC endotherm for the samples containing one major component. The value of Δ*S* at the *T*_m_ value was calculated by the following equation
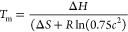
where *c* is the concentration of monomeric peptide, and *R* is the universal gas constant. It was assumed that Δ*C*_p_ was 0 for triple-helix unfolding, and Δ*H* and Δ*S* were independent of temperature.

### Nuclear Magnetic Resonance (NMR) Spectroscopy

NMR experiments were performed on a Bruker Avance-850 NMR spectrometer at the National Tsing Hua University Instrumentation Center. ^1^H,^15^N-HSQC (heteronuclear single quantum coherence) spectra were collected with a total peptide concentration of 0.2 mM in 90% H_2_O/10% D_2_O, pH 7.4, and 20 mM sodium phosphate buffer. For the trimeric state measurements, the peptide solutions were first heated at 80 °C for 20 min, then cooled to room temperature for 15 min, and then incubated at 4 °C for at least 1 week before the collection of NMR spectra. The ^1^H,^15^N-HSQC experiments were conducted at 10 °C to display the trimer resonances. For the monomeric state measurements, the peptide solutions were heated at 80 °C for 20 min to denature the triple helices and then immediately measured at 10 °C without incubation. All the ^1^H,^15^N-HSQC spectra were collected at 10 °C to reduce the noise and increase the resolution. Comparison of different resonance signals at two spectra was then used to identify the formation of the heterotrimer and its composition.

## Results and Discussion

### Evaluation of Various Types of Cation−π Pairwise Effects within a Collagen Triple Helix

Our previous study showed that the introduction of cation−π interactions could stabilize collagen triple helices when the cationic residues were positioned at the Y-site and the aromatic residues at the X-site in the peptide sequence.^23^ However, no stabilization effects could be observed if the relative positions of cationic-aromatic pairs were reversed. Accordingly, we installed cationic Arg residues into the Y-site and aromatic residues (Phe, Tyr, Trp) into the X-site in the designed peptides. Based on the cationic-to-aromatic orientation (as shown in Figure 1), we classified the cation−π interactions within a triple helix into three types: N → C axial pair, C → N axial pair, and lateral pair. To evaluate the contributions of various cation−π pairs, we used (Pro-Hyp-Gly)_8_, POG8, as a parent CMP to design and prepare a set of CMPs based on the arrangements in Figure 1. Sequences of the designed CMPs are listed in Table 1. All the designed CMPs exhibit a sigmoidal thermal unfolding transition as measured by CD (Figures S7–S10 in the Supporting Information), showing that they all form triple helices. The *T*_m_ values determined from CD measurements are summarized in Table 1.

**Table 1:** Peptides Used to Evaluate the Cation−π Pairwise Effects within Collagen Triple Helices

peptide	sequence^a^	*T*_m_ (°C)^b^
POG8	(POG)_8_	50.1 (0.1)
cationic peptides: (POG)*_n_*-P-**Yaa**-G-(POG)_7–*n*_, **Yaa** = **R**		
R4	(POG)_3_-P**R**G-(POG)_4_	48.5 (0.5)
R5	(POG)_4_-P**R**G-(POG)_3_	48.7 (0.3)
aromatic peptides: (POG)_n_-**Xaa**-O-G-(POG)_7–*n*_, **Xaa** = **F**, **Y**, **W**		
R-F series		
F4	(POG)_3_-**F**OG-(POG)_4_	37.4 (0.1)
F5	(POG)_4_-**F**OG-(POG)_3_	37.3 (0.5)
R4F5	(POG)_3_-P**R**G-**F**OG-(POG)_3_	43.4 (0.2)
F4R5	(POG)_3_-**F**OG-P**R**G-(POG)_3_	34.9 (0.2)
F4R4	(POG)_3_-**FR**G-(POG)_4_	28.2 (0.7)
R-Y series		
Y4	(POG)_3_-**Y**OG-(POG)_4_	35.8 (0.2)
Y5	(POG)_4_-**Y**OG-(POG)_3_	36.1 (0.2)
R4Y5	(POG)_3_-P**R**G-**Y**OG-(POG)_3_	43.2 (0.1)
Y4R5	(POG)_3_-**Y**OG-P**R**G-(POG)_3_	33.5 (0.1)
Y4R4	(POG)_3_-**YR**G-(POG)_4_	26.3 (0.2)
R-W series		
W4	(POG)_3_-**W**OG-(POG)_4_	35.1 (0.1)
W5	(POG)_4_-**W**OG-(POG)_3_	34.9 (0.1)
R4W5	(POG)_3_-P**R**G-**W**OG-(POG)_3_	41.1 (0.5)
W4R5	(POG)_3_-**W**OG-P**R**G-(POG)_3_	31.6 (0.2)
W4R4	(POG)_3_-**W*R***G-(POG)_4_	18.9 (0.2)

a All the CMPs had an acetylated N-terminus and an amidated C-terminus.

b The reported *T*_m_ values are the averages of at least two independent measurements, and the deviations are shown in parentheses. The *T*_m_ differences between 4-CMPs and 5-CMPs (R4 vs R5, F4 vs F5, Y4 vs Y5, W4 vs W5) were ≤ 0.3 °C.

Based on the design, we calculated the differences in *T*_m_ values between POG8, monosubstituted cationic CMPs, monosubstituted aromatic CMPs, and cationic aromatic disubstituted CMPs. By using the differences, we could determine the contribution of each type of cation−π pair to the triple helix stability. Meanwhile, we prepared the monosubstituted CMPs on the fourth and fifth triplets (4-CMPs, 5-CMPs), such as R4, R5, F4, and F5, matching the positions substituted in the disubstituted CMPs to make the calculations more accurate. As shown in Table 1, the differences in *T*_m_ values for the CMPs with the same substitution but on the different triplets are relatively small (<0.3 °C). This indicates that using either 4-CMPs or 5-CMPs to determine the impacts of cation−π pairs should be very similar. Since we had synthesized both 4-CMPs and 5-CMPs, we evaluated the impacts of cation−π pairs based on the substitution position.

According to the *T*_m_ values in Table 1, the arrangements in Figure 1, and the diagrams shown in the Supporting Information, we determined the contribution for each type of cation−π pair within a triple helix. Taking the R-Y series as an example, we first calculated the destabilization effects (Δ*T*_m_) for R4, R5, Y4, and Y5 by comparing their *T*_m_ values with that of POG8. Then we calculated the predicted *T*_m_ values for the corresponding CMPs.

The differences in *T*_m_ values between POG8 and the single substitution CMPs (Δ*T*_m_)
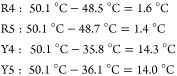


The predicted *T*_m_ values for the double substitution CMPs from the above Δ*T*_m_ values
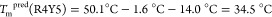

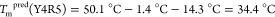

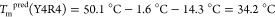


We then compared the observed (as shown in Table 1) and predicted *T*_m_ values for the corresponding CMPs and correlated them with the cation−π pairs shown in Figure 1 to set up the following equations
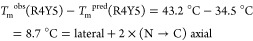
1
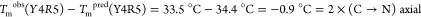
2
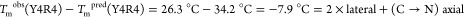
3From eqs 1–3, we determined the impacts of each type of cation−π pair on the triple helix stability—lateral: −3.7 °C, N → C axial: 6.2 °C, C → N axial: −0.45 °C—for the R-Y series of CMPs. Meanwhile, if only the contributions of lateral and N → C axial pairs (eqs 1 and 3) were considered, then their effects on the *T*_m_ value would be lateral: −4.0 °C and N → C axial: 6.3 °C, respectively. Likewise, we determined the impacts for the R-F and R-W series of CMPs (as shown in the Supporting Information). The results indicate that the N → C axial pair stabilizes the triple helix, but the C → N axial and lateral pairs destabilize the structure. Of the three pairwise arrangements, the C → N pair exhibits the smallest effect on the triple helix stability, and in particular, its impact on the R-F and R-Y CMPs is not significant, which affects the *T*_m_ value by less than 1 °C. The contributions of the N → C axial and lateral pairs are similar to those observed by Hartgerink et al.^31^ A recent report showed that the cation−π interaction formed by the R-W pair in water (−4.7 kcal/mol) is more potent than those from the R-F pair (−3.4 kcal/mol) and R-Y pair (−3.7 kcal/mol).^34^ Another recent study using the database of approximately 2700 RCSB Protein Data Bank protein structures revealed that Trp residues occurred more frequently in cation−π interactions than Phe and Tyr residues.^35^ Similarly, our results indicate that the N → C axial stabilization effect in a collagen triple helix is R-W pair > R-Y pair > R-F pair. This further demonstrates that the R-W pair can generate stronger cation−π interactions than R-F and R-Y pairs if the pairwise residues are placed in a suitable orientation and distance while designing peptides. Regarding the unfavorable lateral and C → N axial pairs, the R-W pair has the most destabilization effects among the three pairs, suggesting that the bulky side chain of Trp may induce large steric strains if the cation-aromatic pairs are not in an appropriate location and arrangement. Although the C → N axial pair does not exert as remarkable an effect as the other two pairs on a collagen fold, we have unraveled the impact of the C → N axial pair and provided a more complete picture of the cation−π pairwise effects within a collagen triple helix. The effects of these cation−π pairs on the *T*_m_ values are summarized in Table 2.

**Table 2:** Impacts of Different Types of Cation−π Pairs on the *T*_m_ Values of a Collagen Triple Helix

cationic-aromatic pair		N → C axial (°C)	C → N axial (°C)	lateral (°C)
R-F	a	5.6	–0.55	–3.5
	b	5.8		–3.8
R-Y	a	6.2	–0.45	–3.7
	b	6.3		–4.0
R-W	a	7.3	–1.1	–6.8
	b	7.6		–7.3

a Indicates the effects were determined by considering all the N → C axial, C → N axial, and lateral pairs.

b indicates the effects were determined by considering only the N → C axial and lateral pairs.

### Consequences of Installing Various Axial and Lateral Cation−π Pairs into a Collagen Triple Helix

**Figure 2:**
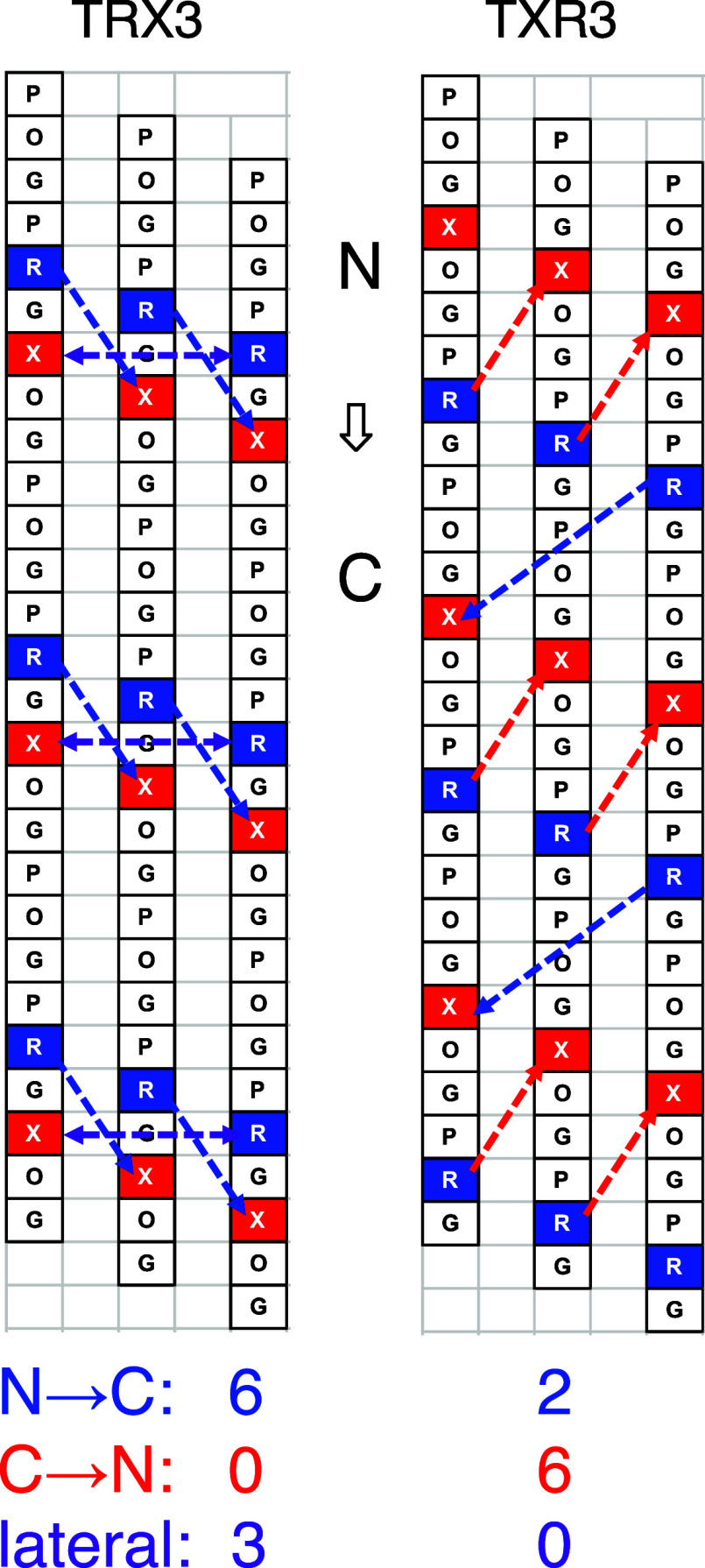
Illustration of cation−π pairs within a collagen homotrimer. The N → C axial pairs are shown in blue arrows, the C → N axial pairs in red arrows, and the lateral pairs in violet arrows. The bottom panel indicates the number of each pair in TRX3 and TXR3.

After revealing that N → C axial, C → N axial, and lateral cation−π pairs have distinct contributions to the collagen triple helix stability, we further used collagen homotrimers to examine if the various types of cation−π pairs incorporated in the CMPs have an additive effect on the trimer stability. Thus, we designed two sets of CMPs: TRX3, [(POG)(PRG)(XOG)]_3_, and TXR3, [(POG)(XOG)(PRG)]_3_, where X is F, Y, or W. The peptides and their sequences used for this study are shown in Table 3. As shown in Figure 2, the homotrimer derived from TRX3 will have six N → C axial pairs and three lateral pairs, while that derived from TXR3 will contain two N → C axial pairs and six C → N axial pairs.

**Table 3:** Sequences of TRX3 and TXR3 and the *T*_m_ Values of Their Triple Helices from CD Measurements

peptide	sequence^a^	*T*_m_ (°C)^b^	Δ*T*_m_^obs^ (°C)	Δ*T*_m_^pred1^ (°C)	Δ_1_ (°C)	Δ*T*_m_^pred2^ (°C)	Δ_2_ (°C)
TRF3	[(POG)(P**R**G)(**F**OG)]_3_	40.1 (0.1)	13.8	15.2	1.4	11.8	–2.0
TFR3	[(POG)(**F**OG)(P**R**G)]_3_	26.3 (0.2)					
TRY3	[(POG)(P**R**G)(**Y**OG)]_3_	40.3 (0.1)	14.0	16.4	2.4	13.2	–0.8
TYR3	[(POG)(**Y**OG)(P**R**G)]_3_	26.3 (0.1)					
TRW3	[(POG)(P**R**G)(**W**OG)]_3_	35.0 (0.1)	12.9	15.4	2.5	8.5	–4.4
TWR3	[(POG)(**W**OG)(P**R**G)]_3_	22.1 (0.1)					

a All the peptides had an acetylated N-terminus and an amidated C-terminus.

b The *T*_m_ values were determined by fitting the unfolding curves into a two-state model. The reported values are the averages of duplicate or triplicate measurements, and the deviations are shown in parentheses. Δ*T*_m_^obs^ = *T*_m_(TRX3) – *T*_m_(TXR3). Δ*T*_m_^pred1^ is the predicted difference in *T*_m_ value of TRX3 and TXR3 by considering all the lateral, N → C axial, and C→N axial pairs, while Δ*T*_m_^pred2^ is that without considering the C → N axial pair. Δ_1_ = Δ*T*_m_^pred1^ – Δ*T*_m_^obs^. Δ_2_ = Δ*T*_m_^pred2^ – Δ*T*_m_^obs^.

**Figure 3:**
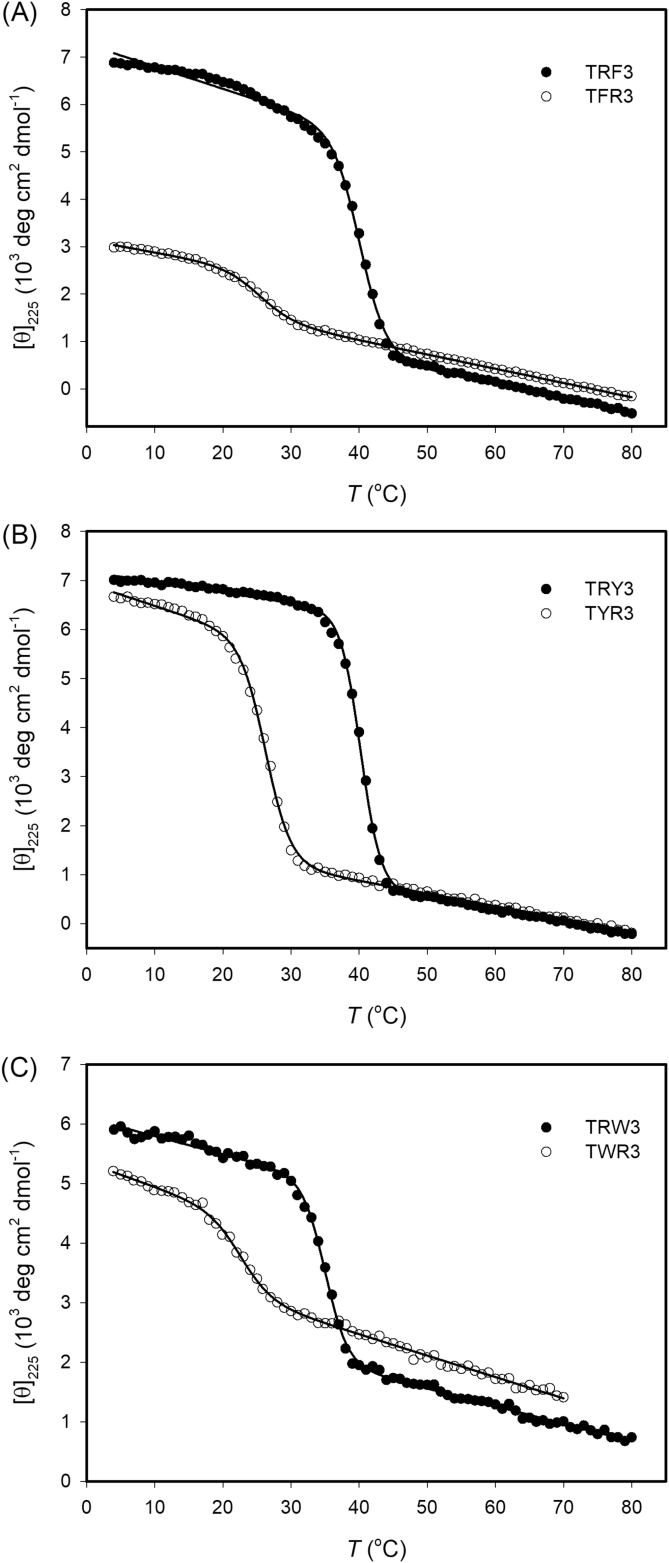
CD-monitored thermal unfolding transitions for TRX3 and TXR3 peptides. All the measurements were conducted in pH 7.0 and 20 mM phosphate buffer with a peptide concentration of 0.2 mM. The heating rate for the measurements was 0.16 °C/min. The solid lines represent the best fit for curves using a two-state model.

Upon synthesizing these designed homotrimeric CMPs, we conducted temperature-dependent CD measurements to determine their thermal stability. As shown in Figure 3, all the peptides exhibit a sigmoidal transition curve, characteristic of a collagen triple helix. Since the triple helices derived from TRX3 peptides contain four more stable N → C axial pairs than TXR3, they have higher *T*_m_ values than the trimers formed by TXR3 peptides, as expected. As indicated in Table 3, the *T*_m_ values of TRF3, TRY3, and TRW3 are more than 10 °C higher than those of their counterpart peptides (TFR3, TYR3, TWR3), indicating that the triple helix stability strongly depends on the number of lateral and axial cation−π pairs. To gain more insight into the additive effects of cation−π pairs on stability, we used the contribution of each type of cation−π pair (Table 2) to calculate the predicted *T*_m_ difference (Δ*T*_m_^pred^) between TRX3 and TXR3. Then we compared the values with the observed values (Δ*T*_m_^obs^). To examine if the C → N axial pair has significant effects on thermal stability, we conducted two different calculations: (1) with all three types of pairs included and (2) with the C → N axial pair excluded. The detailed calculations are shown in the Supporting Information. As shown in Table 3, the deviation (Δ_1_) between Δ*T*_m_^pred1^ and Δ*T*_m_^obs^ is +1.4 to +2.5 °C when all the lateral, N → C axial, and C → N axial cation−π pairs are considered. In contrast, if the C → N axial cation−π pair is excluded from the calculation (Δ*T*_m_^pred2^), the deviation (Δ_2_) will range from −0.8 to −4.4 °C. Comparing these two methods, we found that including the C → N axial pair would overestimate the difference, while excluding it would underestimate the difference. The difference also depends on the cationic-aromatic pairs. The C → N axial pair seems to only slightly affect the prediction on the CMPs with pairwise R-F or R-Y residues; particularly, the impact on R-Y-containing CMPs is minimal. Compared to R-F and R-Y CMPs, the inclusion of the C → N axial pair for R-W CMPs would make the prediction more precise, suggesting that this axial pair might have a larger impact on the CMPs containing Arg and Trp residues. This finding suggests that the contribution of the C → N axial pair may be neglected in the cases of R-F and R-Y paired CMPs but not in R-W paired CMPs.

To further understand the pairwise cation−π interactions in these TRX3 and TXR3 homotrimers, we conducted a simple energy-minimization modeling on the designed sequences and checked the distances between Arg and aromatic residues. As shown in Figures S11 and S12 of the Supporting Information, the average distances for the N → C axial pair, C → N axial pair, and lateral pair are approximately 4.8–7.0, 12.3–14.7, and 5.1–5.2 Å, respectively. The N → C axial distance is similar to that of the R-F pair found in the recently reported crystal structure of a heterotrimer.^33^ From the energy-minimized structure models, the C → N axial pairwise distance is significantly greater than 8 Å, suggesting that this pair is unlikely to generate a considerable interaction. This might explain why we observed its negligible impact on the triple helix stability and why including this pair in the calculation overestimated the predicted Δ*T*_m_ values. Based on the minimized structures, we can also observe that the Arg residues in the lateral and C → N pairs are oriented against the direction of the Arg residues in the N → C pairs. The collagen triple helix has a macrodipole with a positively charged N-terminus and a negatively charged C-terminus. In the lateral and C → N pairs, positively charged Arg residues are aligned against this helix dipole, making these pairs unfavorable in the triple helix. This may also explain why incorporating these two types of pairs into collagen triple helices results in structure destabilization.

**Figure 4:**
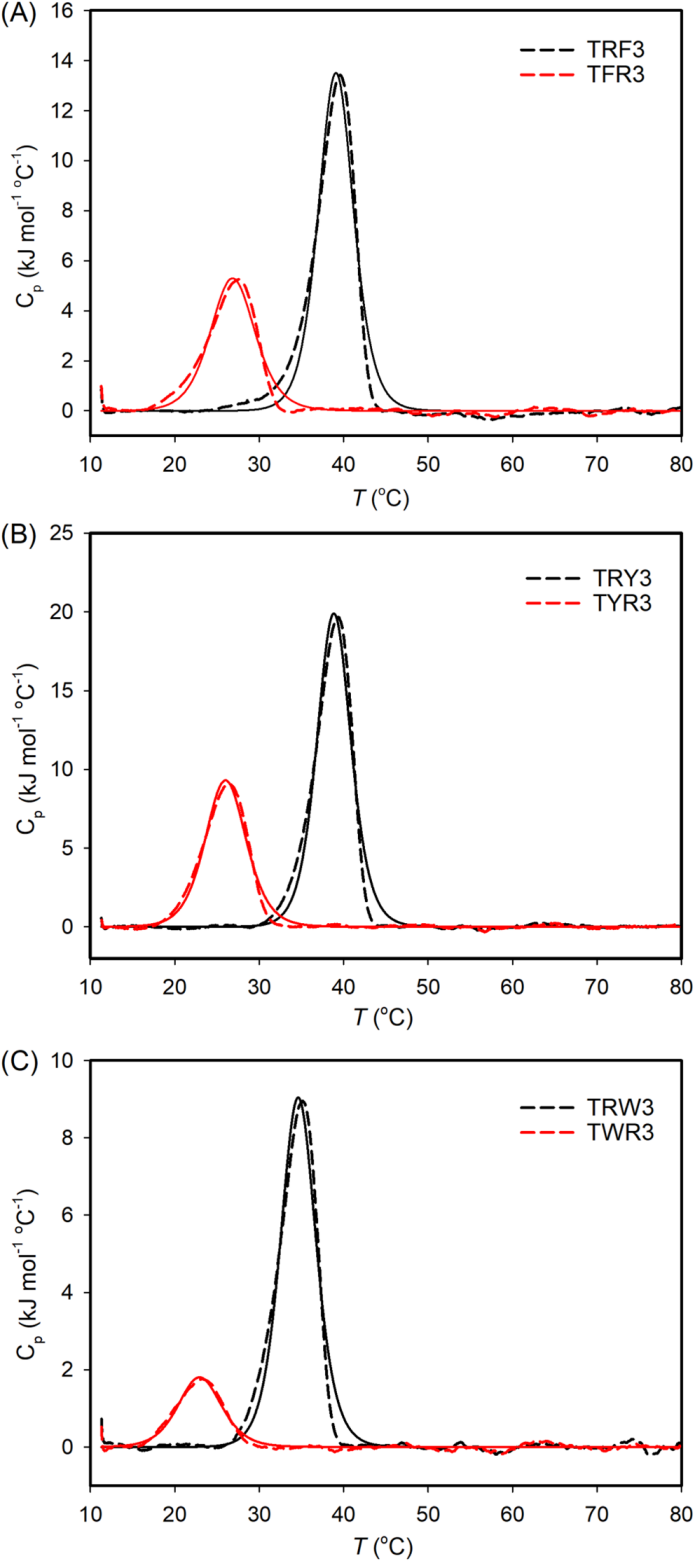
DSC thermograms for the triple helices derived from TRX3 and TXR3 peptides. All the measurements were conducted in pH 7.0 and 20 mM phosphate buffer with a peptide concentration of 0.6 mM. The heating rate for the measurements was 0.1 °C/min. The solid lines represent the best fit for the experimental data (dashed lines).

We also conducted DSC experiments to examine the thermodynamic properties of the homotrimers derived from these CMPs. In Figure 4, the DSC thermograms exhibit one transition for all the peptides, and the thermodynamic parameters obtained from DSC analysis are shown in Table 4. The melting temperatures determined by DSC thermograms are consistent with those from CD measurements. According to DSC analysis, the folding of TRX3 peptides is enthalpy-dominated, while entropic effects mainly control that of TXR3 peptides. The higher stability of the trimers derived from TRX3 peptides than those from TXR3 peptides again supports the assumption that TRX3 CMPs possess more favorable N→C axial cation−π pairs than TXR3 CMPs to facilitate the folding of a trimer.

**Table 4:** Folding Thermodynamic Parameters Derived from DSC Analysis for the Homotrimeric CMPs

peptide	*T*_m_ (°C)	Δ*H* (kJ mol^–1^)	Δ*S* (J mol^–1^ K^–1^)
TRF3	39.1	–75.0	–114.5
TFR3	26.9	–37.3	1.5
TRY3	38.8	–106.5	–215.5
TYR3	26.0	–60.0	–74.9
TRW3	34.6	–50.4	–38.1
TWR3	23.0	–12.4	82.5

### Modulating the Stability of Collagen Heterotrimers by Various Combinations of Lateral and Axial Cation−π Pairs

**Figure 5:**
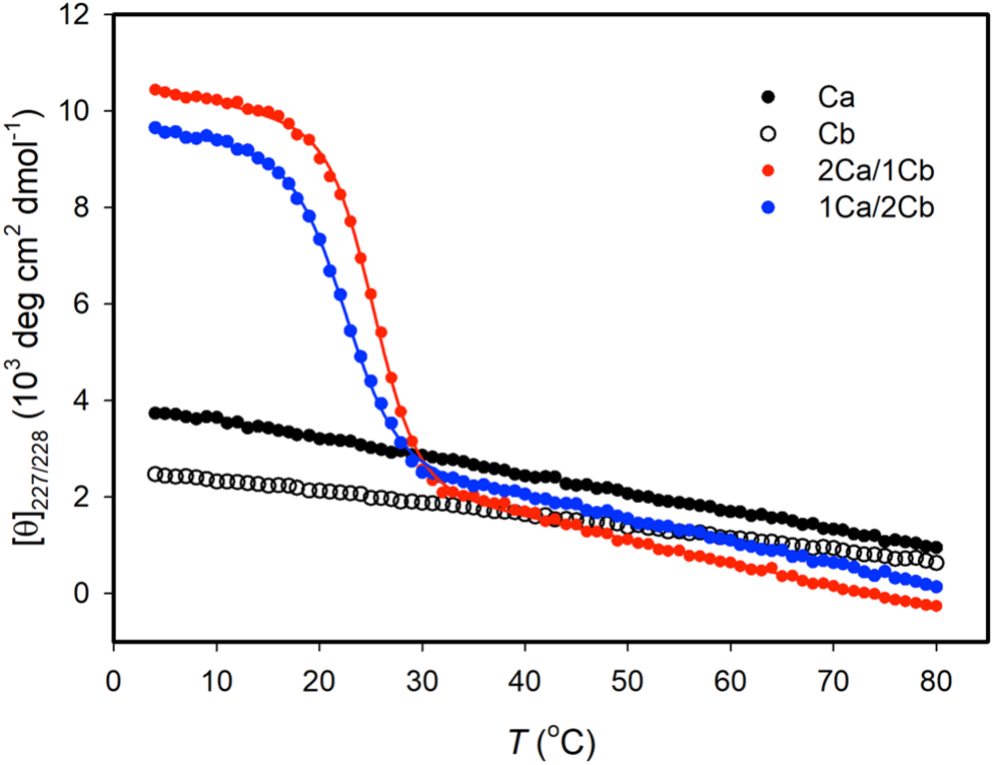
CD-monitored thermal unfolding transitions for Ca, Cb, and their mixtures. All the measurements were conducted in pH 7.4 and 20 mM phosphate buffer with a peptide concentration of 0.2 mM. The heating rate for the measurements was 0.16 °C/min. The solid lines represent the best fit for curves using a two-state model.

Our previous studies showed that cation−π interactions could induce the formation of AAB-type collagen heterotrimers.^21,22^ In those studies, we did not consider the contributions of lateral cation−π pairs and axial cation−π pairs individually. Since we learned that the lateral pair, the N → C axial pair, and the C → N axial pair induce dramatically distinct effects on the triple helix stability, we intended to examine the impacts of implanting various cation−π pairs on the heterotrimeric folds. Accordingly, we used the R-Y pair as an example to design and synthesize the two CMPs, as shown in Table 5, to study cation−π interaction-induced collagen heterotrimers. Like the CMPs used in the homotrimer study, Ca and Cb peptides contained an acetylated N-terminus and an amidated C-terminus. After we prepared the peptides, we conducted CD measurements on the solution of the Ca, Cb, and Ca/Cb mixture. Figure 5 shows that Ca and Cb do not exhibit sigmoidal thermal unfolding curves, indicating that these two CMPs do not fold into triple helices. In contrast to the individual Ca and Cb, the mixture of Ca and Cb with a molar ratio of two-to-one (2Ca/1Cb) or one-to-two (1Ca/2Cb) displays a sigmoidal transition curve, showing the formation of heterotrimers. We took the first derivatives of the CD signals versus temperature, which gave only one minimum for each mixture (Figure S13 in the Supporting Information), indicating that only one heterotrimeric component exists in the solution. The *T*_m_ values obtained from the first derivatives are 26 °C for 2Ca/1Cb and 23 °C for 1Ca/2Cb, respectively, suggesting that the mixtures with various molar ratios fold into different types of heterotrimers via the cation−π interactions within the triple helix. The *T*_m_ values determined by using a two-state model to fit the unfolding curves are 25.3 °C for 2Ca/1Cb and 22.8 °C for 1Ca/2Cb (Table 5), consistent with those from taking the first derivatives of CD signals versus temperature.

**Table 5:** Peptides Used for Heterotrimeric Folding, and the *T*_m_ Values Measured by CD

peptide	sequence	*T*_m_ (°C)^b^
Ca	(PRG)_4_-(POG)-(YOG)_4_	no trimers
Cb	(YOG)_4_-(POG)-(PRG)_4_	no trimers
2Ca/1Cb^a^		25.3 (0.1)
1Ca/2Cb		22.8 (0.3)

a 2Ca/1Cb indicates the molar ratio of Ca to Cb is 2 to 1. 1Ca/2Cb indicates the molar ratio of Ca to Cb is 1 to 2.

b The reported *T*_m_ values are the averages of duplicate measurements. The values in the parentheses indicate the deviations.

**Figure 6:**
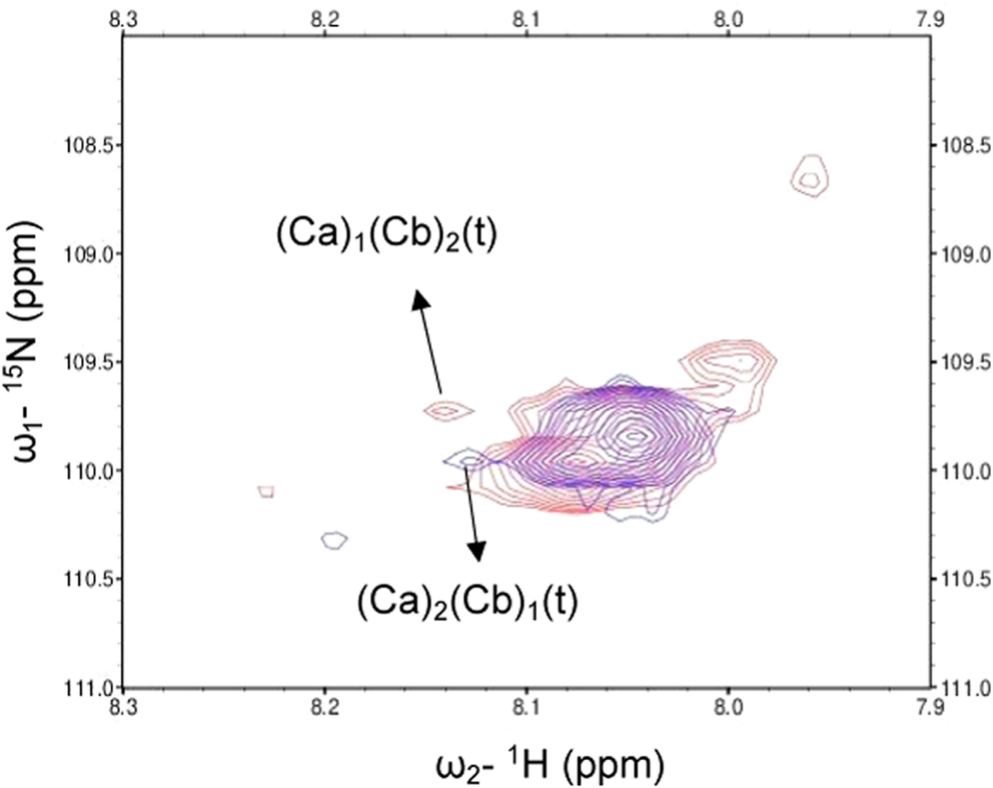
Overlapped ^1^H, ^15^N–HSQC spectra of 2Ca/1Cb (violet) and 1Ca/2Cb (magenta) at 10 °C. The (*t*) denotes trimers for the heterotrimers. Ca* and Cb* peptides were used in the measurements.

To further investigate if the heterotrimer formed in the 2Ca/1Cb solution is different from that formed in the 1Ca/2Cb solution, we prepared Ca* and Cb* with the middle Gly residue in the sequence substituted by ^15^N enriched Gly (G*), i.e., Ca*: (PRG)_4_-(POG*)-(YOG)_4_ and Cb*: (YOG)_4_-(POG*)-(PRG)_4_ to perform ^1^H,^15^N-HSQC NMR experiments. In the NMR experiments, we first took individual HSQC spectra at low and high temperatures for Ca* and Cb* peptides to assign the peaks corresponding to monomers and trimers. Since Ca and Cb peptides do not form triple helices, we only observed the peaks for their monomers (Figure S14 in the Supporting Information). Likewise, we then recorded HSQC spectra on 2Ca*/1Cb* and 1Ca*/2Cb* at low and high temperatures. The HSQC spectra were then overlapped and compared to check if there were new peaks in addition to the signals from the monomers of Ca and Cb. As shown in Figure S14 of the Supporting Information, there is a peak that is not from the monomers of either Ca or Cb in the 2Ca/1Cb and 1Ca/2Cb samples, indicating the formation of heterotrimers. Furthermore, Figure 6 shows the overlapped spectra of the 2Ca/1Cb and 1Ca/2Cb samples, which reveal two different trimer peaks, providing evidence that mixtures with varied molar ratios of Ca to Cb generate different AAB-type heterotrimers. Thus, the heterotrimer formed in the 2Ca/1Cb mixture was designated (Ca)_2_(Cb)_1_, while that formed in the 1Ca/2Cb mixture was designated (Ca)_1_(Cb)_2_. From HSQC experiments, we confirmed that mixtures of Ca and Cb form AAB-type heterotrimers, whose composition depends on the molar ratio of Ca to Cb.

According to the CD measurements, the *T*_m_ value of the (Ca)_2_(Cb)_1_ trimer is higher than that of the (Ca)_1_(Cb)_2_ trimer, suggesting that the cation−π interaction in the (Ca)_2_(Cb)_1_ trimer is stronger than that in the (Ca)_1_(Cb)_2_ trimer. We then compared the number of lateral and axial cation−π pairs between these two heterotrimers by using all possible strand register assemblies in a heterotrimer (Figure S15 in the Supporting Information). From the assemblies, it is clear that (Ca)_2_(Cb)_1_ and (Ca)_1_(Cb)_2_ contain the same number of lateral cation−π pairs and C → N axial cation−π pairs in each strand register, while (Ca)_2_(Cb)_1_ has one more N → C axial cation−π pair in the register of Ca·Cb·Ca than that of (Ca)_1_(Cb)_2_ in the register of Cb·Ca·Cb (Table 6). As shown in Table 2, the N → C axial cation−π pair has a positive contribution to the trimer stability, and thus (Ca)_2_(Cb)_1_ is expected to form a more stable trimer than (Ca)_1_(Cb)_2_. This is consistent with the experimental results from CD and suggests that the folds of (Ca)_2_(Cb)_1_ and (Ca)_1_(Cb)_2_ might follow the Ca·Cb·Ca and Cb·Ca·Cb registers, respectively. Furthermore, the *T*_m_ difference between (Ca)_2_(Cb)_1_ and (Ca)_1_(Cb)_2_ is 2.5 °C by CD, which is smaller than the stabilization effect of one N → C axial R-Y pair (+6.2 °C), suggesting that the correlation to the stability cannot simply count on the number of cation−π pairs in these two heterotrimers. This implies that a heterotrimeric fold is more complicated than a homotrimeric one, and more factors must be considered. Nonetheless, the observations from cation−π interaction-induced heterotrimers further support the idea that varying the lateral and axial cation−π pairs can modulate the folding stability of a heterotrimer. The formation of a collagen heterotrimer from designed CMPs could be controlled by increasing the favorable N → C axial cation−π pairs and decreasing the unfavorable lateral and C → N axial cation−π pairs.

**Table 6:** Number of Cation−π Pairs in (Ca)_2_(Cb)_1_ and (Ca)_1_(Cb)_2_ for Three Possible Strand Register Assemblies

register type (lead·middle·lag)	lateral pair	N → C axial pair	C → N axial pair
(Ca)_2_(Cb)_1_			
Ca·Ca·Cb	7	5	7
Ca·Cb·Ca	8	7	6
Cb·Ca·Ca	7	5	7
(Ca)_1_(Cb)_2_			
Cb·Cb·Ca	7	5	7
Cb·Ca·Cb	8	6	6
Ca·Cb·Cb	7	5	7

## Conclusions

Cation−π interactions have been recognized as critical forces in stabilizing protein structures,^36^ and their physiochemical characteristics fit well in the hydrophobic and hydrophilic environments of membrane proteins,^37^ indicating their significant roles in biological systems. We previously showed that such interactions could be used to stabilize a collagen triple helix and induce the formation of AAB-type collagen heterotrimers.^21,22^ In this study, we used a series of CMPs to investigate the impacts of different pairwise cation−π arrangements, including lateral pairs, N → C axial pairs, and C → N axial pairs, on the collagen triple helix stability. Our results indicate that only the N → C axial pair stabilizes the triple helix, while the C → N axial pair and the lateral pair impose a destabilization effect. We also found that the impact of the C → N axial cation−π pair is relatively small, except for the Arg-Trp pair, providing useful information for designing cation−π interactions to stabilize collagen trimers. Using the impacts of lateral and N → C axial cation−π pairs on the melting temperature of a collagen homotrimer, we have shown that the predictions of *T*_m_ values with pairwise Arg-Phe and Arg-Tyr residues incorporated are relatively close to the experimental data. However, the effect of the C → N axial pair needs to be considered to better predict the *T*_m_ values of pairwise Arg-Trp residue-incorporated CMPs. Additionally, we also used cation−π interaction-induced AAB-type collagen heterotrimers to demonstrate that increasing the favorable N → C axial cation−π pairs could form a more stable heterotrimer. The results show that combining various orientations of cation−π pairs can modulate the triple helix stability and be used to design desired CMPs. In conclusion, we have revealed more insights into the contributions of the three types of pairwise cation−π arrangements within a collagen triple helix and demonstrated their use to predict and regulate triple helix stability, which is valuable in designing collagen-related materials.

## Supporting Information Available

Methods for calculating the effects of different cation−π pairs in *T*_m_ values; occurrence frequency of Arg-aromatic pairs in collagen sequences; molecular weights of the peptides used in this study; HPLC chromatograms; CD-monitored thermal unfolding curves for POG8, RF, RY, and RW series of CMPs; plots of the first derivatives of unfolding curves for heterotrimers; additional ^1^H,^15^N-HSQC spectra; and the strand arrangements in different registers are included (PDF)
