# Supplementary material for: Evaluation of Orientation-Dependent Cation−π Pairwise Effects within Collagen Triple Helices
Source: J Phys Chem B. 2025 Apr 30;129(19):4605–13. doi: 10.1021/acs.jpcb.4c08691 (PMC12086834; doi:10.1021/acs.jpcb.4c08691)
Supplement: Supplementary file 1 — jp4c08691_si_001.pdf [file jp4c08691_si_001.pdf]

Supporting Information for

Evaluation of Orientation-Dependent Cation–π Pairwise Effects within Collagen Triple Helices

Tzu-Jou Yao[a], Yung-En Ke[a], Wen-Ling Lin[a], You-Cheng Lin[a], Chih-Han Yang[a], Tsai-Ling Hsu[a], and Jia-Cherng Horng[a,b]*

[a]Department of Chemistry, National Tsing Hua University, Hsinchu, Taiwan 300044, R.O.C.

[b]Frontier Research Center on Fundamental and Applied Sciences of Matters, National Tsing Hua University, Hsinchu, Taiwan 300044, R.O.C.

Table of Contents

(A) The procedure to calculate the contributions of cation–π pairs	S2
(B) Calculations of ΔT_m^{pred1} and ΔT_m^{pred2}	S4
**Table S1.** The frequencies of pairwise Arg-aromatic sequences found in the common human collagens (types I, II, III, IV)	S5
**Table S1.** Molecular weights for the peptides used in this work	S6
**Figure S1.** Analytical HPLC chromatograms for the CMPs used to determine the contribution of individual cation–π pairs in this study.	S7-S9
**Figure S2.** Analytical HPLC chromatograms for peptides TRF3 and TFR3.	S10
**Figure S3.** Analytical HPLC chromatograms for peptides TRY3 and TYR3.	S11
**Figure S4.** Analytical HPLC chromatograms for peptides TRW3 and TWR3.	S12
**Figure S5.** Analytical HPLC chromatograms for peptides TRF3 and TFR3.	S13
**Figure S6.** Analytical HPLC chromatograms for peptides TRF3 and TFR3.	S14
**Figure S7.** Thermal unfolding transitions for POG8, R4, and R5.	S15
**Figure S8.** Thermal unfolding transitions for F4, F5, R4F5, F4R5, and F4R4.	S16
**Figure S9.** Thermal unfolding transitions for Y4, Y5, R4Y5, Y4R5, and Y4R4.	S17
**Figure S10.** Thermal unfolding transitions for W4, W5, R4W5, W4R5, and W4R4.	S18
**Figure S11.** The energy-minimized structure models for TRX3 CMPs	S19
**Figure S12.** The energy-minimized structure models for TXR3 CMPs	S20
**Figure S12.** Plots of the first derivatives of molar ellipticity versus temperature for the thermal unfolding curves of 2Ca/1Cb and 1Ca/2Cb solutions.	S21
**Figure S13.** ^{15}N-^{1}H HSQC spectra for Ca, Cb, and their mixtures in different molar ratios.	S22
**Figure S14.** Different strand arrangements for the heterotrimers $(Ca)_2(Cb)_1$ and $(Ca)_1(Cb)_2$.	S23

(A) The procedure to calculate the contribution of different types of cation–π pairs for R-F and R-W CMPs:

The following diagrams show the interactions corresponding to the designed CMPs, where X is F, Y, or W.

R4X5: 2 × (N→C axial) + 1× (lateral) pairs

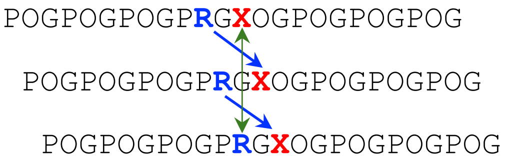

X4R5: 2 × (C→N axial) pairs

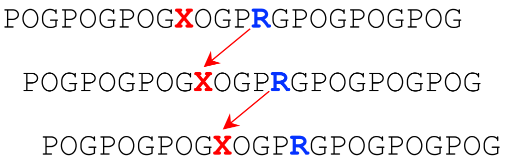

X4R4: 1 × (C→N axial) + 2× (lateral) pairs

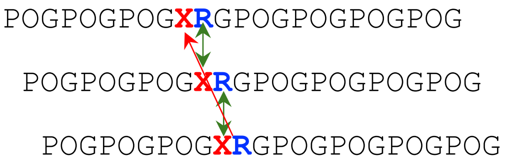

I. R-F series:

The differences in T_m values between POG8 and the single substitution CMPs (ΔT_m):

R4: 50.1 °C – 48.5 °C = 1.6 °C

R5: 50.1 °C – 48.7 °C = 1.4 °C

F4: 50.1 °C – 37.4 °C = 12.7 °C

F5: 50.1 °C – 37.3 °C = 12.8 °C

The predicted T_m values for the double substitution CMPs from the above ΔT_m values:

T_m^{pred}(R4F5) = 50.1 °C – 1.6 °C – 12.8 °C = 35.7 °C

T_m^{pred}(F4R5) = 50.1 °C – 1.4 °C – 12.7 °C = 36.0 °C

T_m^{pred}(F4R4) = 50.1 °C – 1.6 °C – 12.7 °C = 35.8 °C

The observed and predicted T_m values for the corresponding CMPs were compared and correlated with the cation–π pairs shown in Figure 1 to set up the following equations.

(a) With all the N→C axial, C→N axial, and lateral pairs included:

T_m^{obs}(R4F5) – T_m^{pred}(R4F5) = 43.4 °C – 35.7 °C = 7.7 °C = lateral + 2 × (N→C) axial (1)

T_m^{obs}(F4R5) – T_m^{pred}(F4R5) = 34.9 °C – 36.0 °C = –1.1 °C = 2 × (C→N) axial (2)

T_m^{obs}(F4R4) – T_m^{pred}(F4R4) = 28.2 °C – 35.8 °C = –7.6 °C = 2 × lateral + (C→N) axial (3)

⇒ N→C axial: 5.6 °C; C→N axial: –0.55 °C; lateral: –3.5 °C

(b) With only the N→C axial and lateral pairs included:

T_m^{obs}(R4F5) – T_m^{pred}(R4F5) = 43.4 °C – 35.7 °C = 7.7 °C = lateral + 2 × (N→C) axial (1)

T_m^{obs}(F4R4) – T_m^{pred}(F4R4) = 28.2 °C – 35.8 °C = –7.6 °C = 2 × lateral (2)

⇒ N→C axial: 5.8 °C; lateral: –3.8 °C

II. R-W series:

The differences in T_m values between POG8 and the single substitution CMPs (ΔT_m):

R4: 50.1 °C – 48.5 °C = 1.6 °C

R5: 50.1 °C – 48.7 °C = 1.4 °C

W4: 50.1 °C – 35.1 °C = 15.0 °C

W5: 50.1 °C – 34.9 °C = 15.2 °C

The predicted T_m values for the double substitution CMPs from the above ΔT_m values:

T_m^{pred}(R4W5) = 50.1 °C – 1.6 °C – 15.2 °C = 33.3 °C

T_m^{pred}(W4R5) = 50.1 °C – 1.4 °C – 15.0 °C = 33.7 °C

T_m^{pred}(W4R4) = 50.1 °C – 1.6 °C – 15.0 °C = 33.5 °C

The observed and predicted T_m values for the corresponding CMPs were compared and correlated with the cation–π pairs shown in Figure 1 to set up the following equations.

(a) With all the N→C axial, C→N axial, and lateral pairs included:

T_m^{obs}(R4W5) – T_m^{pred}(R4W5) = 41.1 °C – 33.3 °C = 7.8 °C = lateral + 2 × (N→C) axial (1)

T_m^{obs}(W4R5) – T_m^{pred}(W4R5) = 31.6 °C – 33.7 °C = –2.1 °C = 2 × (C→N) axial (2)

T_m^{obs}(W4R4) – T_m^{pred}(W4R4) = 18.9 °C – 33.5 °C = –14.6 °C = 2 × lateral + (C→N) axial (3)

⇒ N→C axial: 7.3 °C; C→N axial: –1.1 °C; lateral: –6.8 °C

(b) With only the N→C axial and lateral pairs included:

T_m^{obs}(R4W5) – T_m^{pred}(R4W5) = 41.1 °C – 33.3 °C = 7.8 °C = lateral + 2 × (N→C) axial (1)

T_m^{obs}(W4R4) – T_m^{pred}(W4R4) = 18.9 °C – 33.5 °C = –14.6 °C = 2 × lateral + (C→N) axial (2)

⇒ N→C axial: 7.6 °C; C→N axial: –1.1 °C; lateral: –7.3 °C

(B) Calculations of ΔT_m^{pred1} and ΔT_m^{pred2}

TRF3 vs. TFR3:

ΔT_m^{pred1}: including all three cation–π pairs

TRF3 – TFR3 = [(N→C axial) × 6 + lateral × 3]– [(N→C axial) × 2 + (C→N axial) × 6] = (5.6 × 6 – 3.5 × 3) – (5.6 × 2 – 0.55 × 6) = 15.2

ΔT_m^{pred2}: excluding the C→N axial pairs

TRF3 – TFR3 = [(N→C axial) × 6 + lateral × 3]– (N→C axial) × 2 = (5.8 × 6 – 3.8 × 3) – (5.8 × 2) = 11.8

TRY3 vs. TYR3:

ΔT_m^{pred1}: including all three cation–π pairs

TRY3 – TYR3 = [(N→C axial) × 6 + lateral × 3]– [(N→C axial) × 2 + (C→N axial) × 6] = (6.2 × 6 – 3.7 × 3) – (6.2 × 2 – 0.45 × 6) = 16.4

ΔT_m^{pred2}: excluding the C→N axial pairs

TRY3 – TYR3 = [(N→C axial) × 6 + lateral × 3]– (N→C axial) × 2 = (6.3 × 6 – 4.0 × 3) – (6.3 × 2) = 13.2

TRW3 vs. TWR3:

ΔT_m^{pred1}: including all three cation–π pairs

TRW3 – TWR3 = [(N→C axial) × 6 + lateral × 3]– [(N→C axial) × 2 + (C→N axial) × 6] = (7.3 × 6 – 6.8 × 3) – (7.3 × 2 – 1.1 × 6) = 15.4

ΔT_m^{pred2}: excluding the C→N axial pairs

TRW3 – TWR3 = [(N→C axial) × 6 + lateral × 3]– (N→C axial) × 2 = (7.6 × 6 – 7.3 × 3) – (7.6 × 2) = 8.5

Table S1. The frequencies of pairwise Arg-aromatic sequences found in the common human collagens (types I, II, III, IV).

Collagen Chain[a]	Number of Occurrences				
	RGF	RGY	GFR	GYR	FX'GY'RG[b]
α1[I]	5	0	0	0	1
α2[I]	1	1	0	0	1
α1[II]	5	1	0	0	1
α1[III]	2	0	1	0	1
α1[IV]	5	1	0	1	2
α2[IV]	1	1	3	1	3

[a]Types I and IV are heterotrimers while types II and III are homotrimers.
[b]X' and Y' represent any amino acids.
The sequences are from www.unitprot.org

Table S2. Molecular weights for the peptides used in this work

Peptide	Calculated mass (M)	Observed mass
POG8	2196.01	2220.35 [M+Na$^+$]
R4	2239.06	2240.82 [M+H$^+$]
R5	2239.06	2240.21 [M+H$^+$]
F4	2246.02	2269.19 [M+Na$^+$]
F5	2246.02	2269.35 [M+Na$^+$]
R4F5	2289.07	2290.12 [M+H$^+$]
F4R5	2289.07	2290.94 [M+H$^+$]
F4R4	2289.07	2290.12 [M+H$^+$]
Y4	2262.01	2284.54 [M+Na$^+$]
Y5	2262.01	2284.79 [M+Na$^+$]
R4Y5	2305.07	2305.72 [M+H$^+$]
Y4R5	2305.07	2306.78 [M+H$^+$]
Y4R4	2305.07	2307.90 [M+H$^+$]
W4	2285.03	2309.71 [M+Na$^+$]
W5	2285.03	2309.32 [M+Na$^+$]
R4W5	2328.08	2329.20 [M+H$^+$]
W4R5	2328.08	2329.60 [M+H$^+$]
W4R4	2328.08	2329.21 [M+H$^+$]
TRF3	2742.34	2743.84 [M+H$^+$]
TFR3	2742.34	2743.16 [M+H$^+$]
TYR3	2790.33	2791.20 [M+H$^+$]
TRY3	2790.33	2791.41 [M+H$^+$]
TWR3	2859.37	2861.55 [M+H$^+$]
TRW3	2859.37	2861.62 [M+H$^+$]
Ca	2899.41	2900.09 [M+H$^+$]
Cb	2899.41	2900.58 [M+H$^+$]
Ca*	2900.41	2901.11 [M+H$^+$]
Cb*	2900.41	2901.15 [M+H$^+$]

(A) POG8

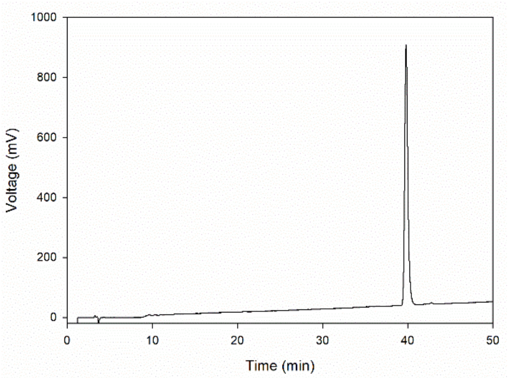

(B) R4

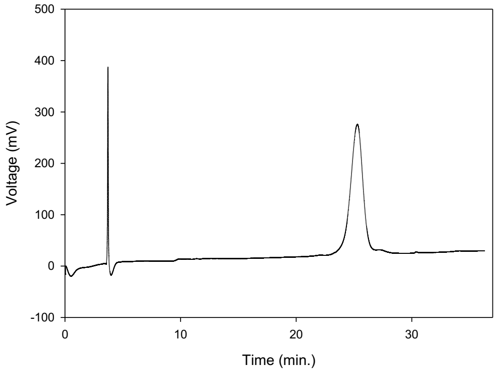

(C) R5

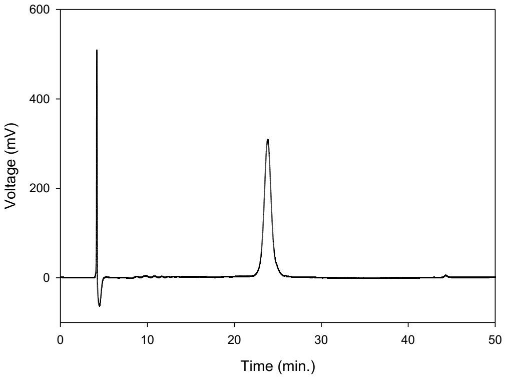

(D) F4

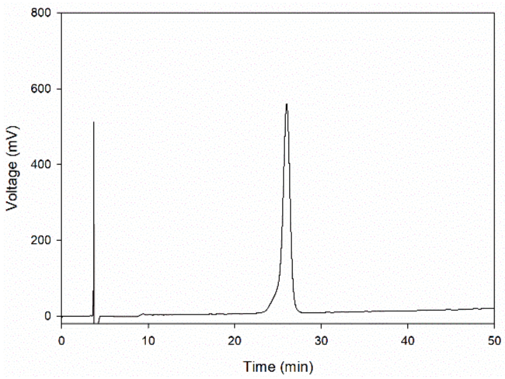

(E) F5

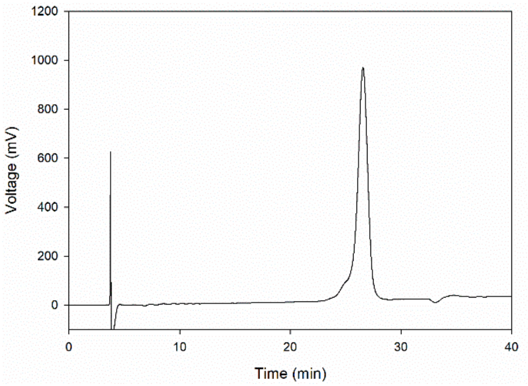

(F) Y4

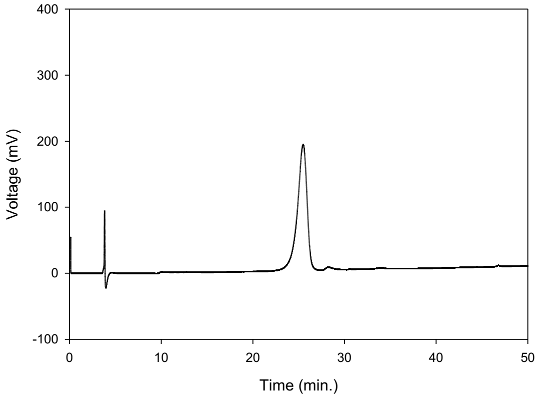

(G) Y5

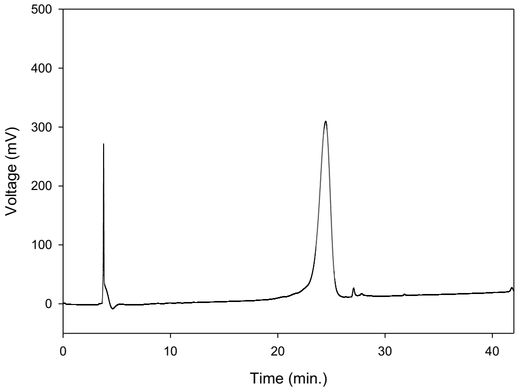

(H) W4

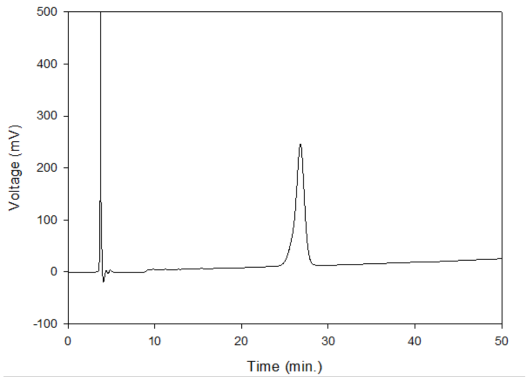

(I) W5

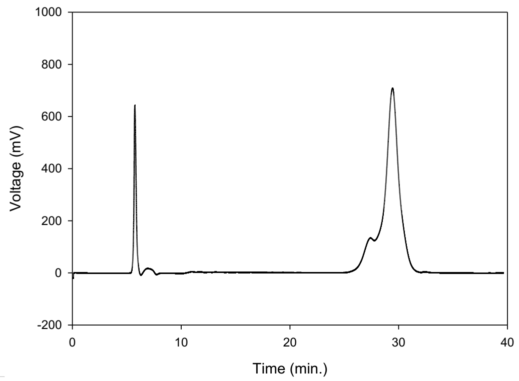

(J) R4F5

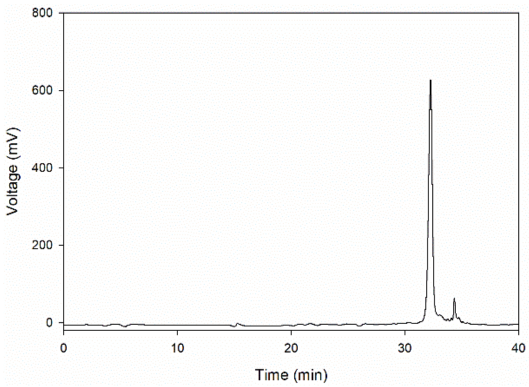

(K) F4R5

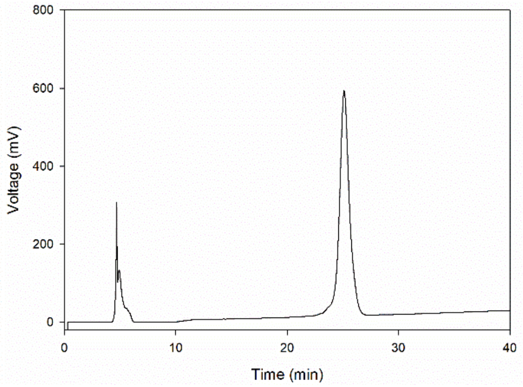

(L) F4R4

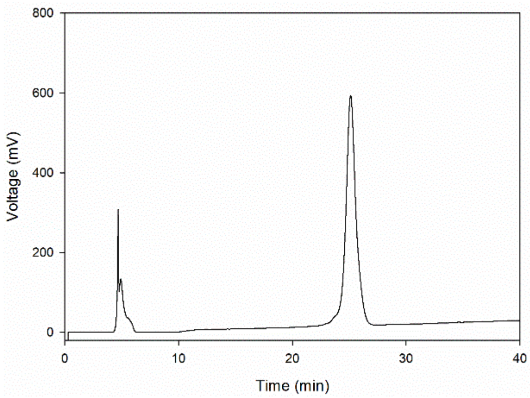

(M) R4Y5

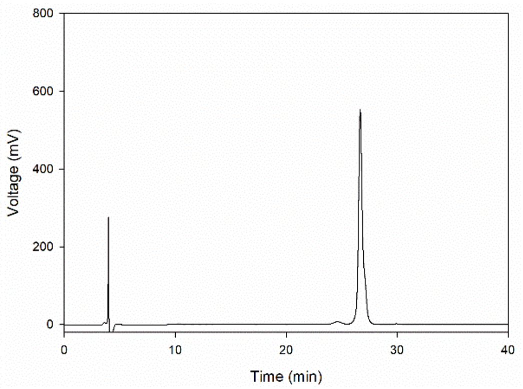

(N) Y4R5

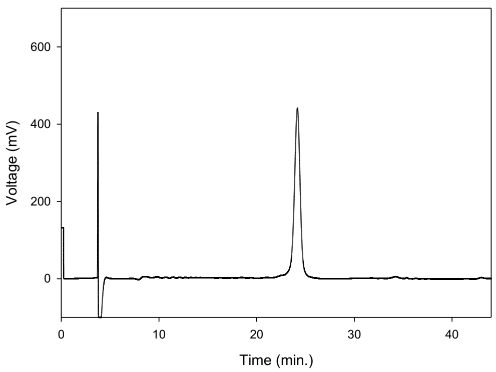

(O) Y4R4

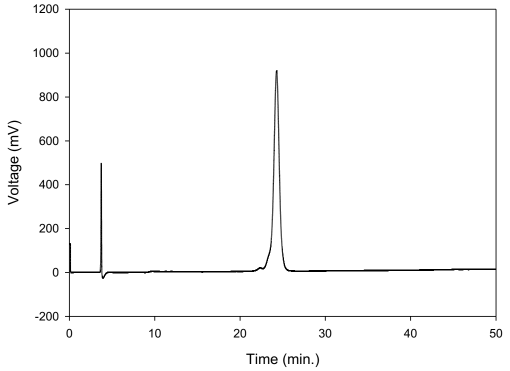

(P) R4W5

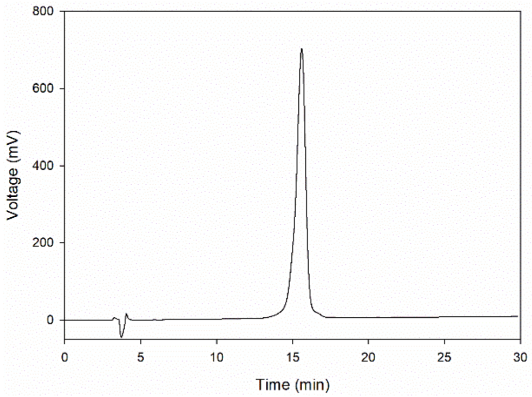

(Q) W4R5

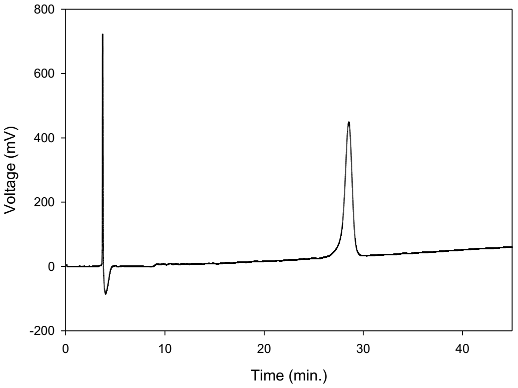

(R) W4R4

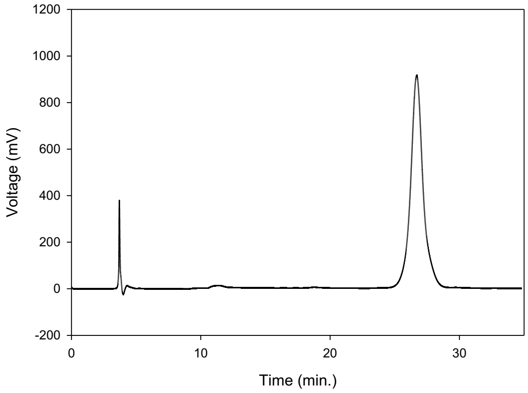

Figure S1. Analytical HPLC chromatograms for the CMPs used to determine the contribution of individual cation–π pairs in this study. The experiments were conducted using a C18 analytic column with a gradient of 0% to 50% acetonitrile containing 0.1% (v/v) TFA in 50 min. The peaks before 10 min were due to the solvent used to dissolve the peptides.

(A) TRF3

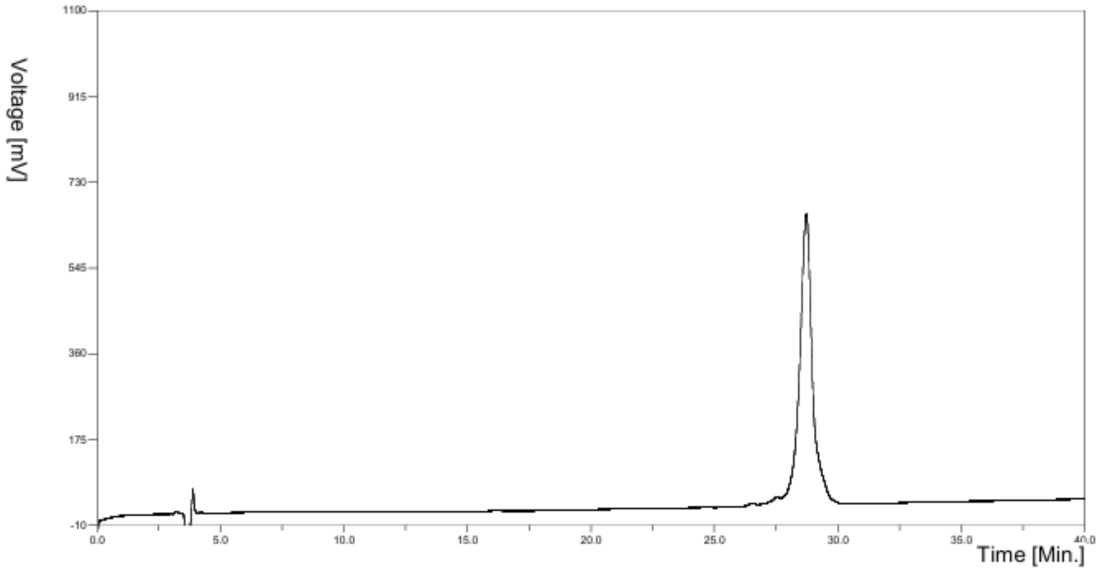

(B) TFR3

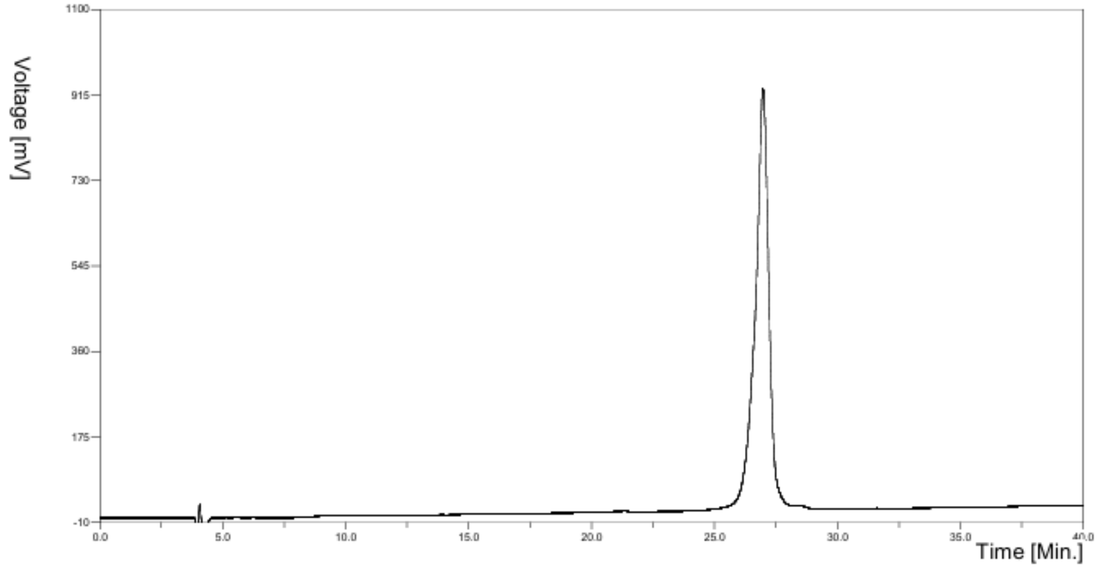

Figure S2. Analytical HPLC chromatograms for peptides TRF3 and TFR3. The experiments were conducted using a C18 analytic column with a gradient of 0% to 50% acetonitrile containing 0.1% (v/v) TFA in 50 min. The peaks before 5 min were due to the solvent used to dissolve the peptides.

(A) TRY3

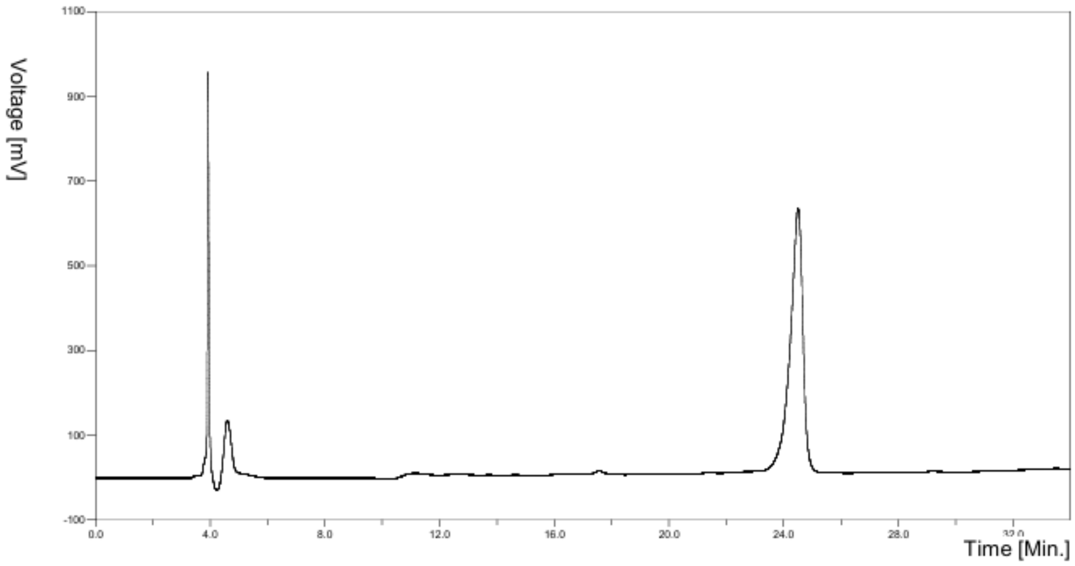

(B) TYR3

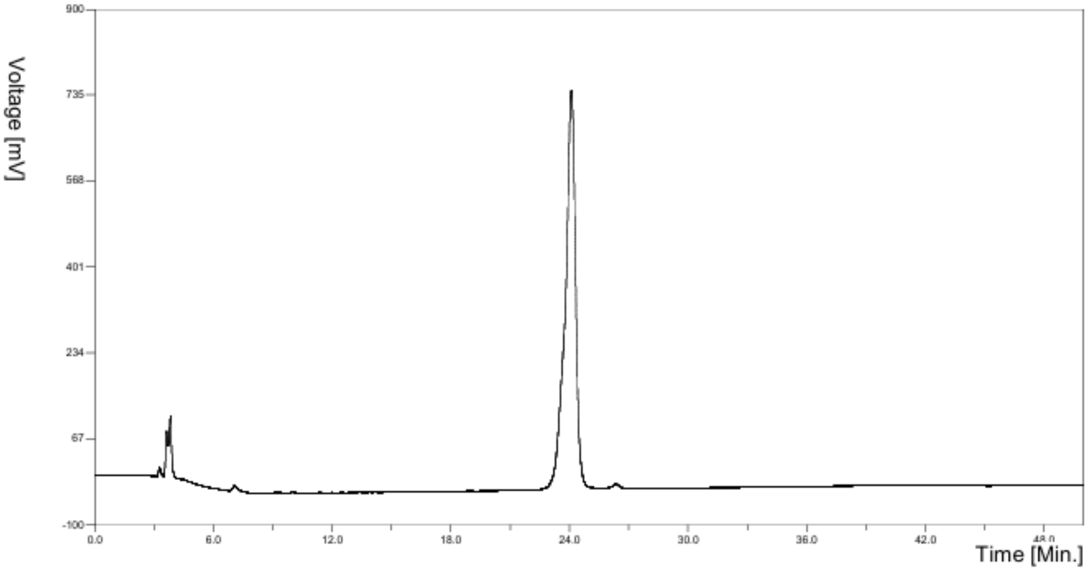

Figure S3. Analytical HPLC chromatograms for peptides TRY3 and TYR3. The experiments were conducted using a C18 analytic column with a gradient of 0% to 50% acetonitrile containing 0.1% (v/v) TFA in 50 min. The peaks before 10 min were due to the solvent used to dissolve the peptides.

(A) TRW3

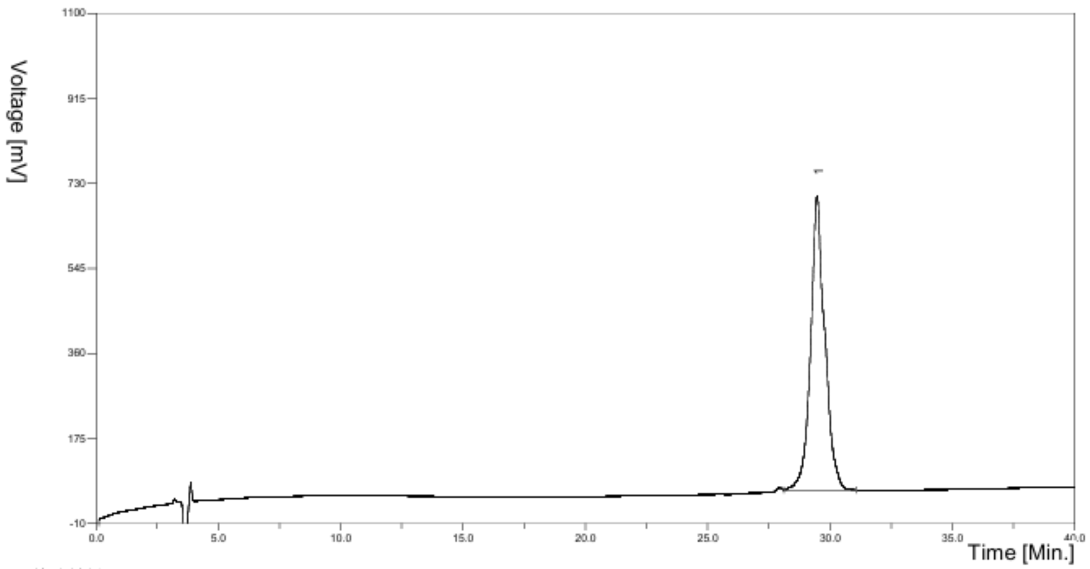

(B) TWR3

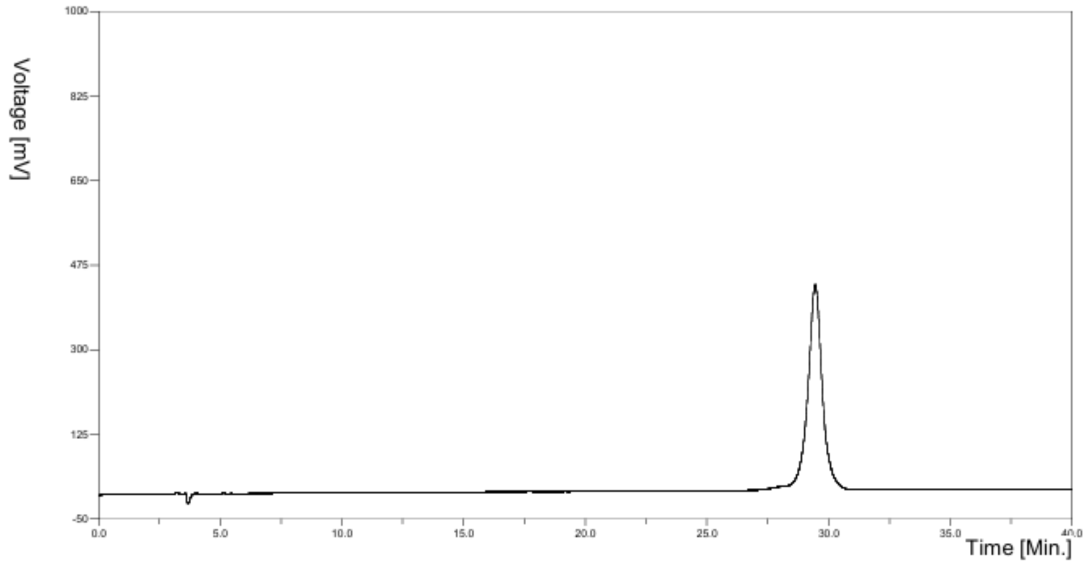

Figure S4. Analytical HPLC chromatograms for peptides TRW3 and TWR3. The experiments were conducted using a C18 analytic column with a gradient of 0% to 50% acetonitrile containing 0.1% (v/v) TFA in 50 min. The peaks before 5 min were due to the solvent used to dissolve the peptides.

(A) Ca

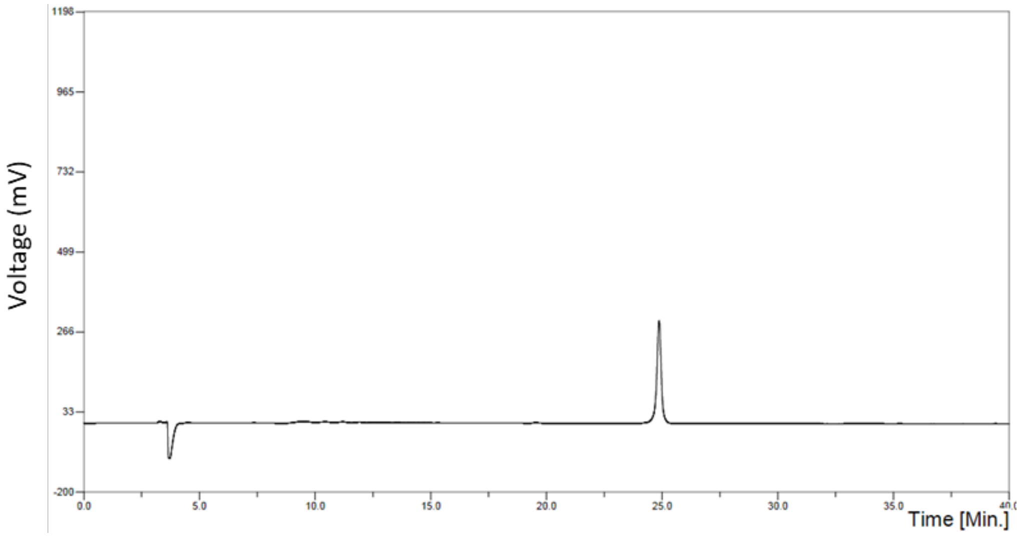

(B) Cb

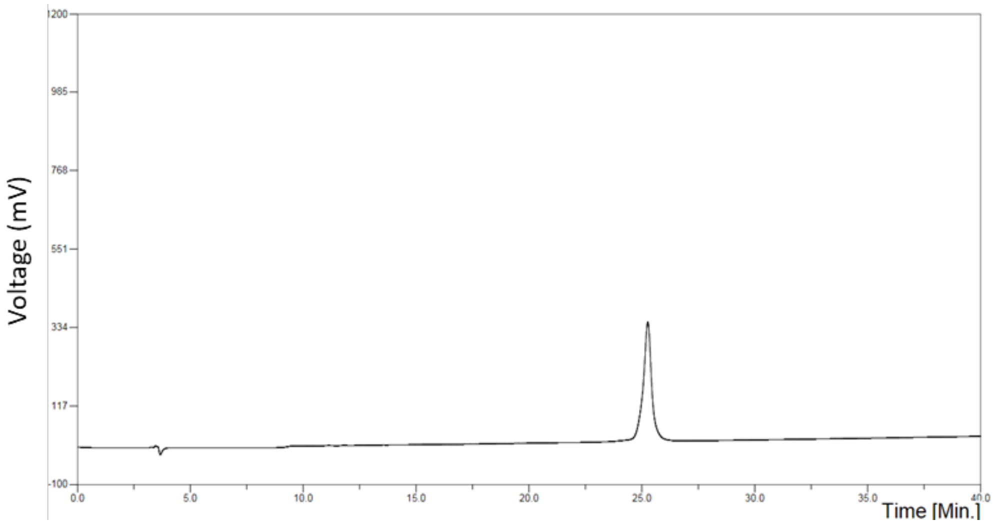

Figure S5. Analytical HPLC chromatograms for peptides Ca and Cb. The experiments were conducted using a C18 analytic column with a gradient of 0% to 50% acetonitrile containing 0.1% (v/v) TFA in 50 min. The peaks before 5 min were due to the solvent used to dissolve the peptides.

(A) Ca*

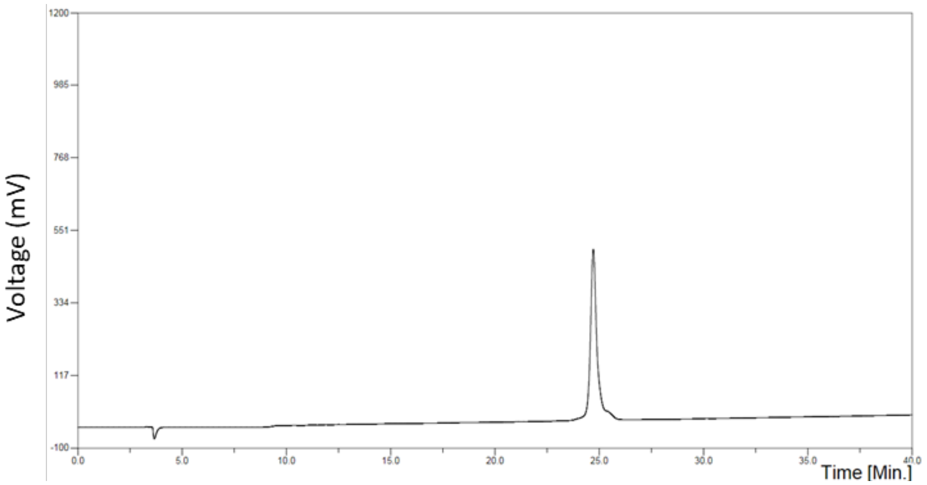

(B) Cb*

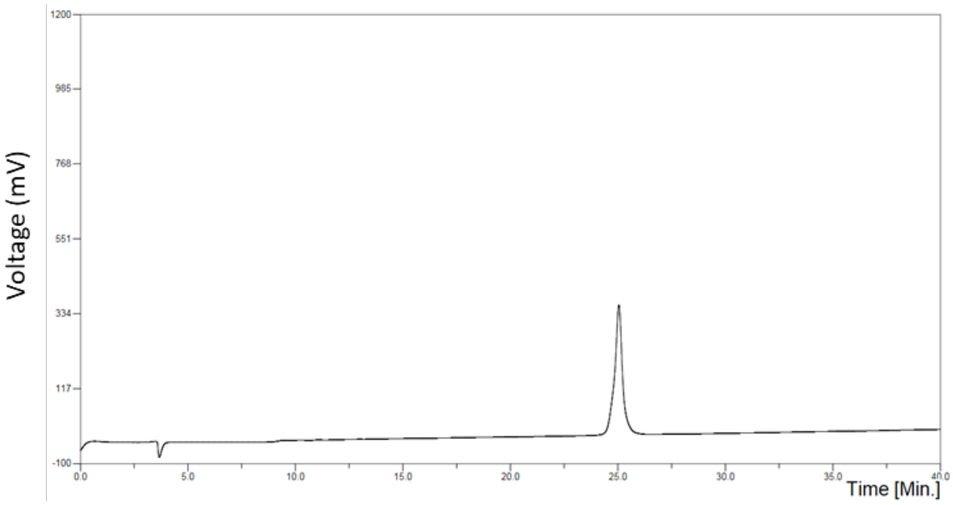

Figure S6. Analytical HPLC chromatograms for peptides Ca* and Cb*. The experiments were conducted using a C18 analytic column with a gradient of 0% to 50% acetonitrile containing 0.1% (v/v) TFA in 50 min. The peaks before 5 min were due to the solvent used to dissolve the peptides.

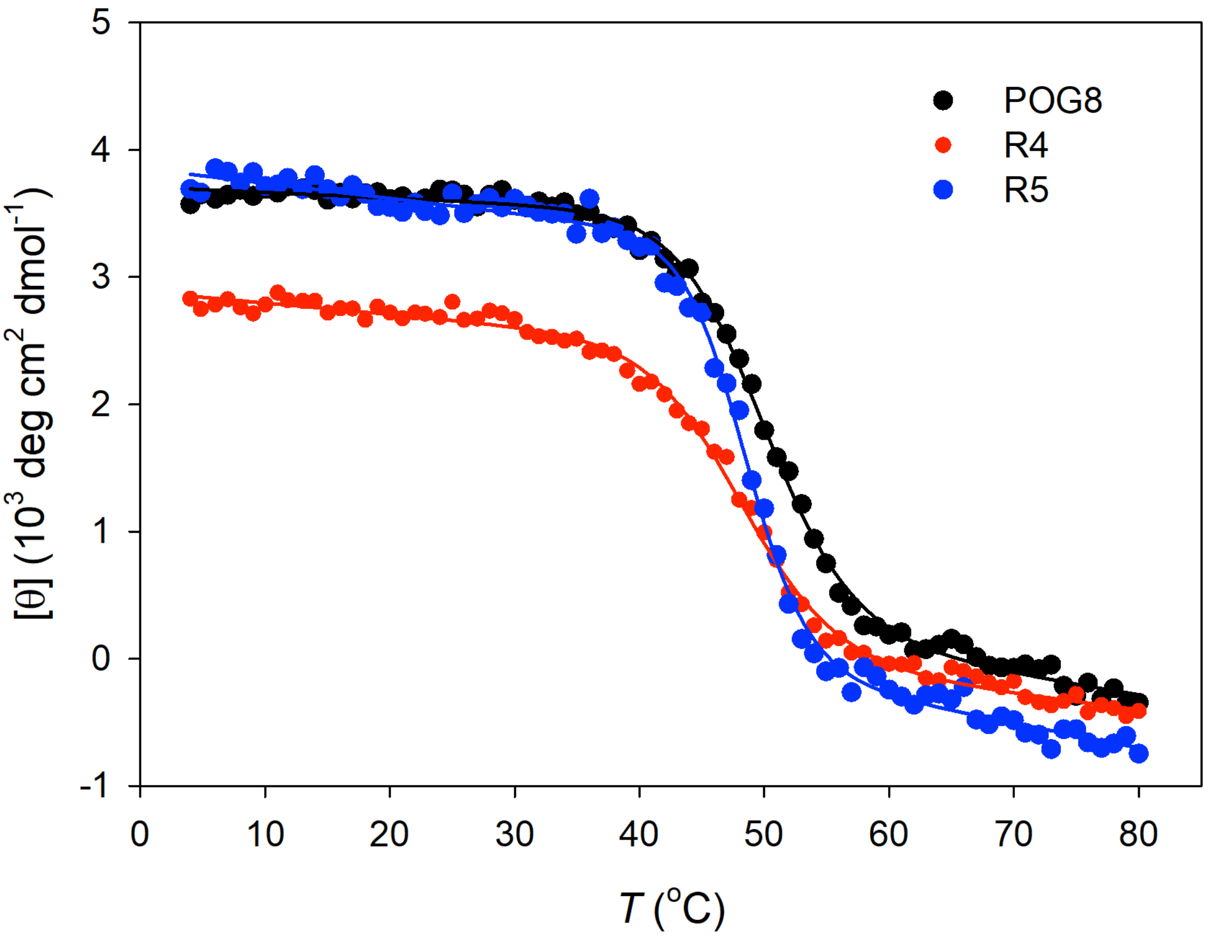

Figure S7. Thermal unfolding transitions for POG8, R4, and R5. All the measurements were conducted in pH 7.0 and 20 mM phosphate buffer with a peptide concentration of 0.2 mM. The solid lines indicate the best fit for the data using a two-state model.

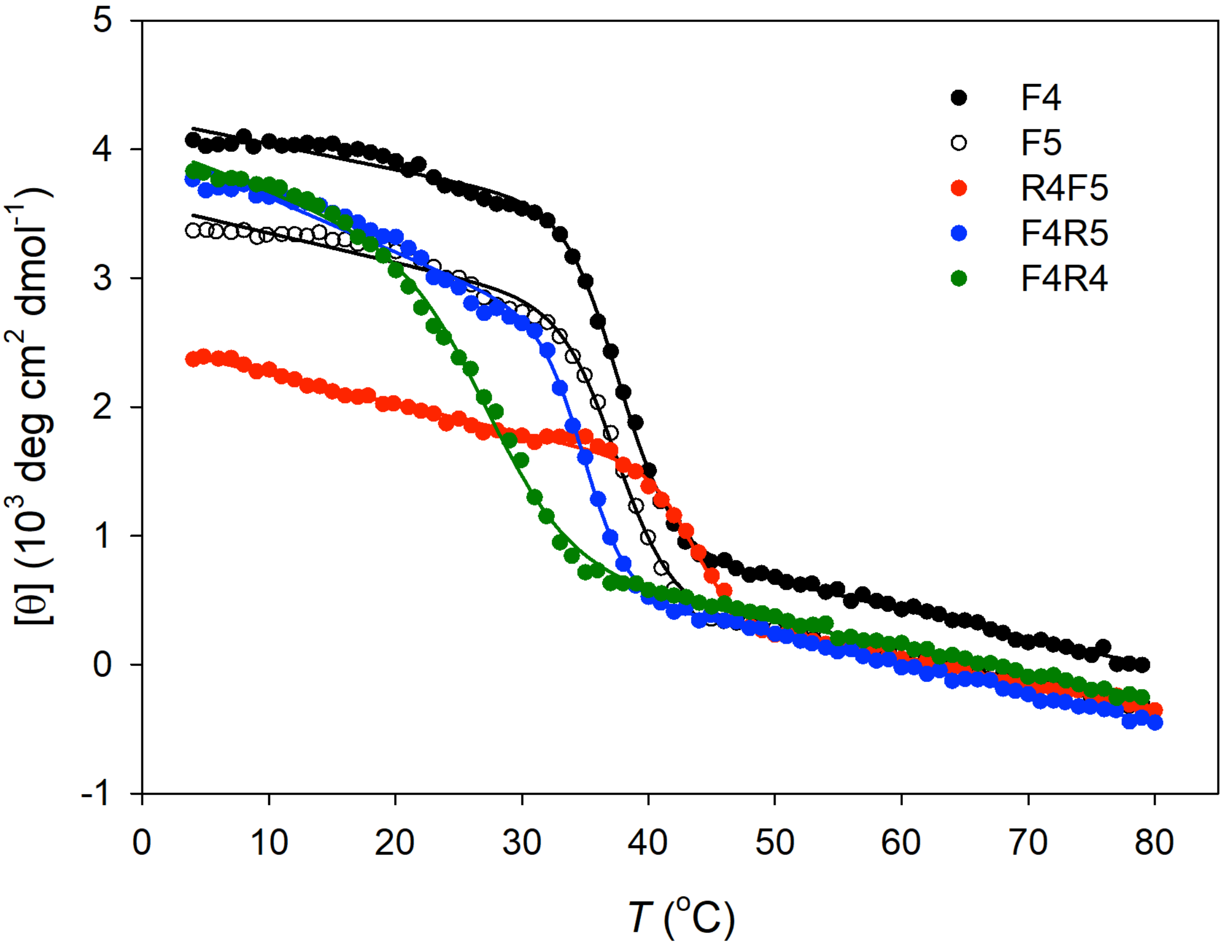

Figure S8. Thermal unfolding transitions for F4, F5, R4F5, F4R5, and F4R4. All the measurements were conducted in pH 7.0 and 20 mM phosphate buffer with a peptide concentration of 0.2 mM. The solid lines indicate the best fit for the data using a two-state model.

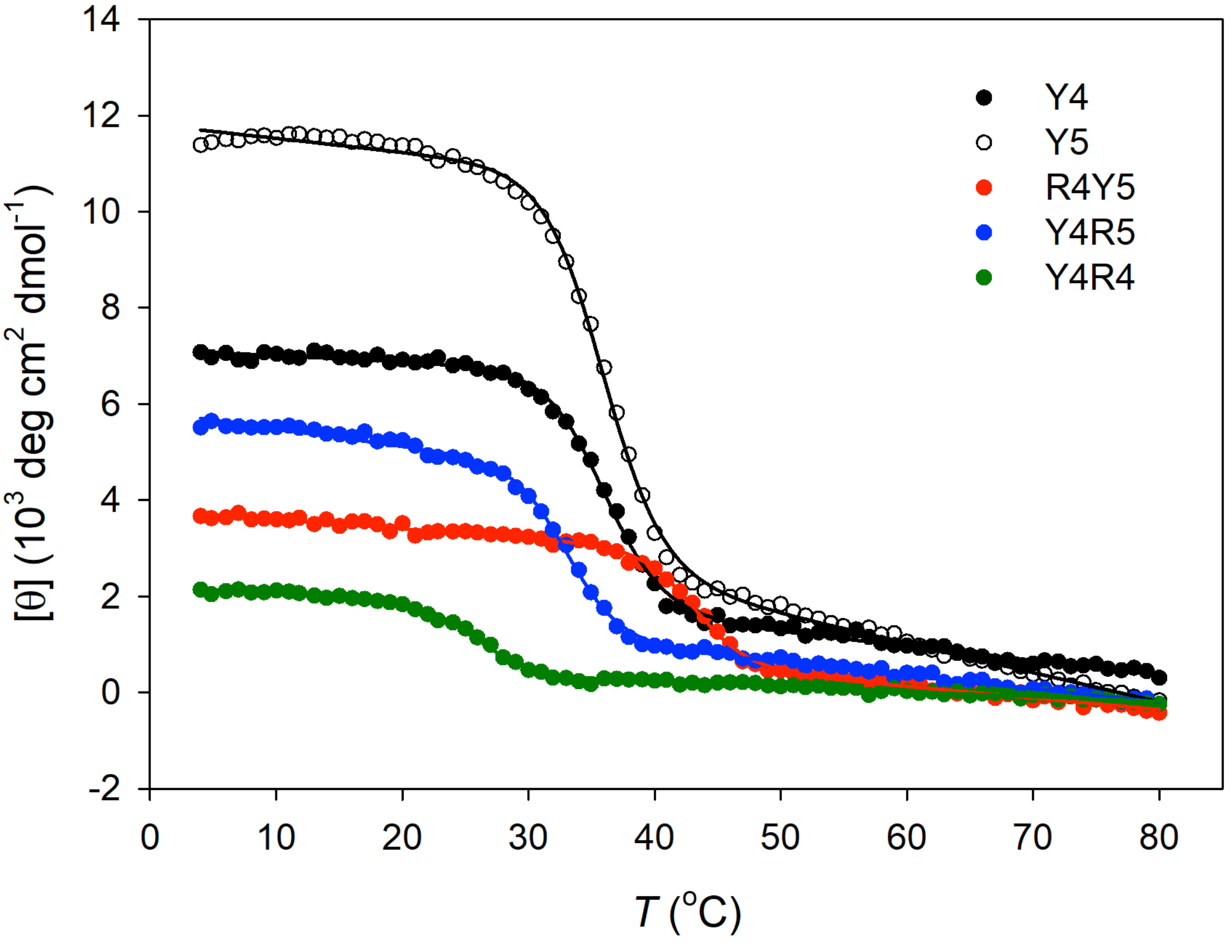

Figure S9. Thermal unfolding transitions for Y4, Y5, R4Y5, Y4R5, and Y4R4. All the measurements were conducted in pH 7.0 and 20 mM phosphate buffer with a peptide concentration of 0.2 mM. The solid lines indicate the best fit for the data using a two-state model.

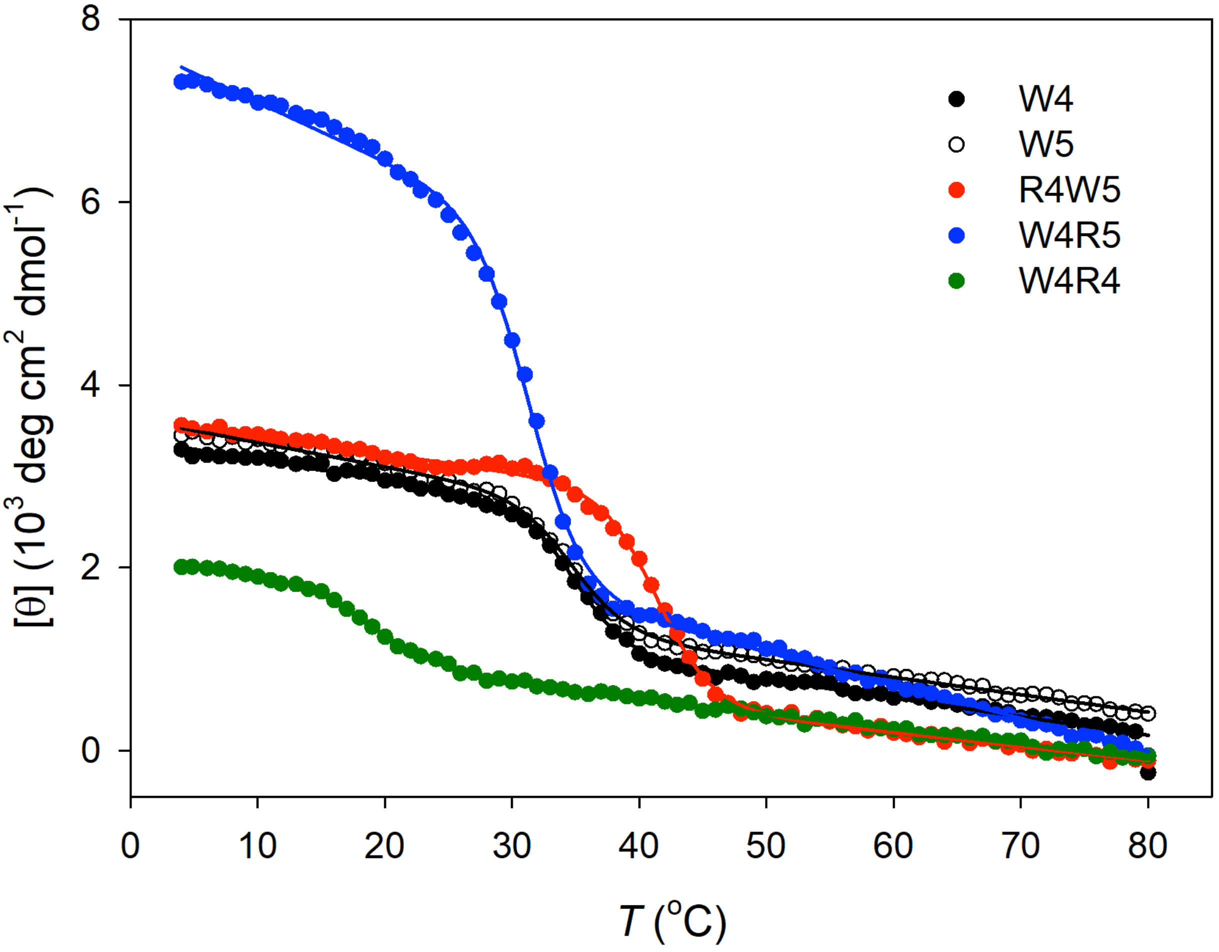

Figure S10. Thermal unfolding transitions for W4, W5, R4W5, W4R5, and W4R4. All the measurements were conducted in pH 7.0 and 20 mM phosphate buffer with a peptide concentration of 0.2 mM. The solid lines indicate the best fit for the data using a two-state model.

(A) TRF3: [(POG)(PRG)(FOG)]$_3$

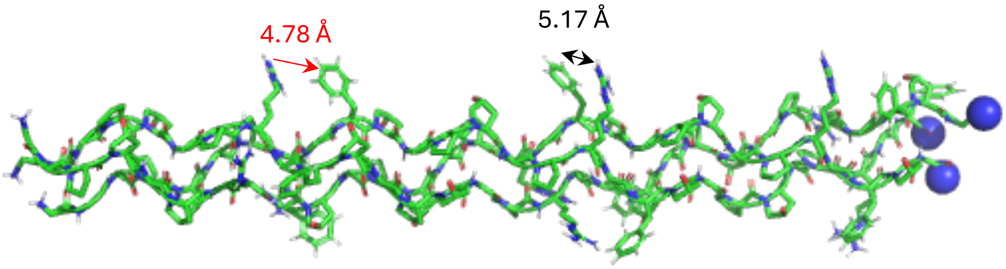

(C) TRY3: [(POG)(PRG)(YOG)]$_3$

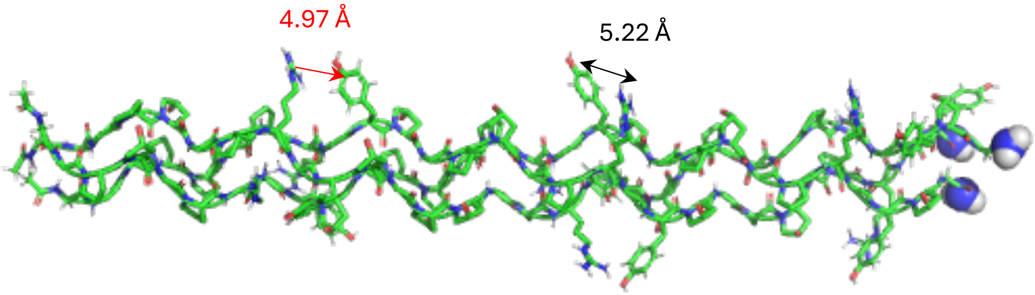

(D) TRW3: [(POG)(PRG)(WOG)]$_3$

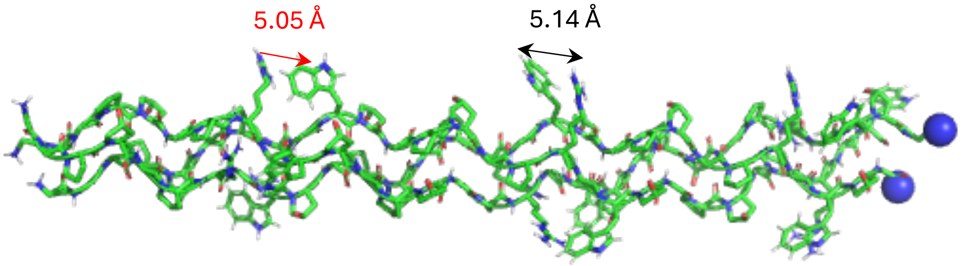

Figure S11. The energy-minimized structure models for TRX3 CMPs are (A) TRF3, (B) TRY3, and (C) TRW3. The PDB file 2DRX was used as the template, and Arg and aromatic residues were incorporated into the Xaa and Yaa positions, respectively. Then, the structure models were generated in the SPDBV program (https://spdbv.unil.ch) to conduct energy minimization. The distances between Arg and aromatic sidechains were measured using the Gaussian View program, and the average values are shown in the diagrams. The N→C axial pairs are in red, and the lateral pairs are in black. Only one pairwise distance is presented for each type of pair.

(A) TFR3: [(POG)(FOG)(PRG)]$_3$

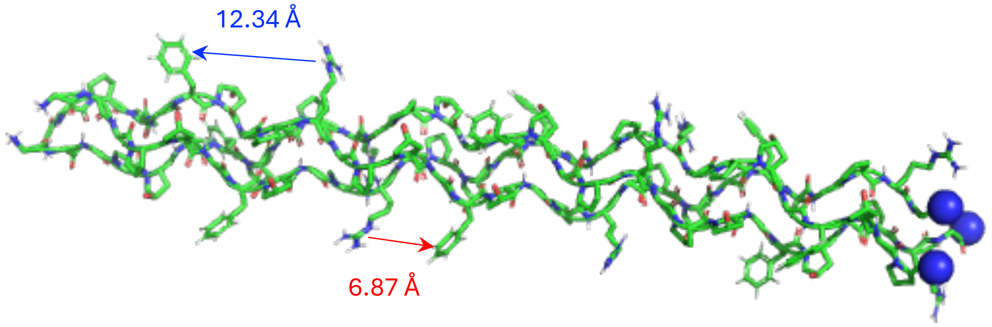

(B) TYR3: [(POG)(YOG)(PRG)]$_3$

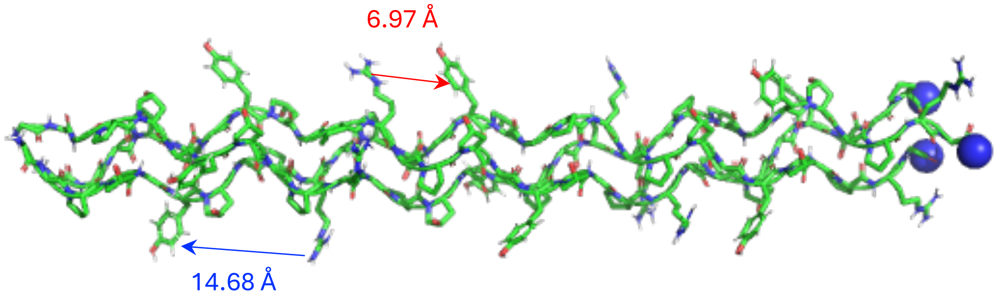

(C) TWR3: [(POG)(WOG)(PRG)]$_3$

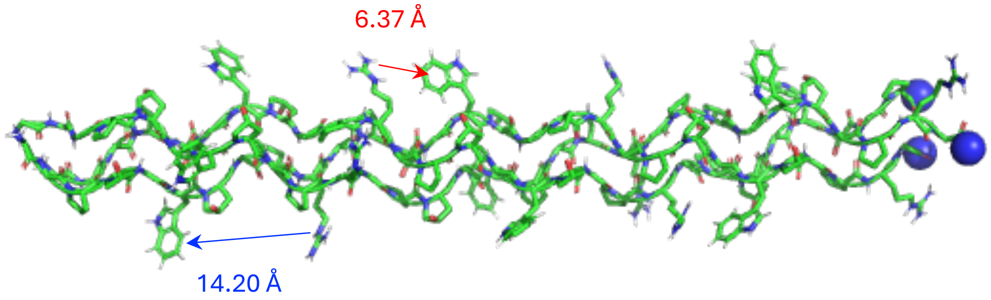

Figure S12. The energy-minimized structure models for TXR3 CMPs are (A) TFR3, (B) TYR3, and (C) TWR3. The PDB file 2DRX was used as the template, and R and aromatic residues were incorporated into the Xaa and Yaa positions, respectively. Then, the structure models were generated in the SPDBV program (https://spdbv.unil.ch) to conduct energy minimization. The distances between Arg and aromatic sidechains were measured using the Gaussian View program, and the average values are shown in the diagrams. The N→C axial pairs are in red, and the C→N pairs are in blue.

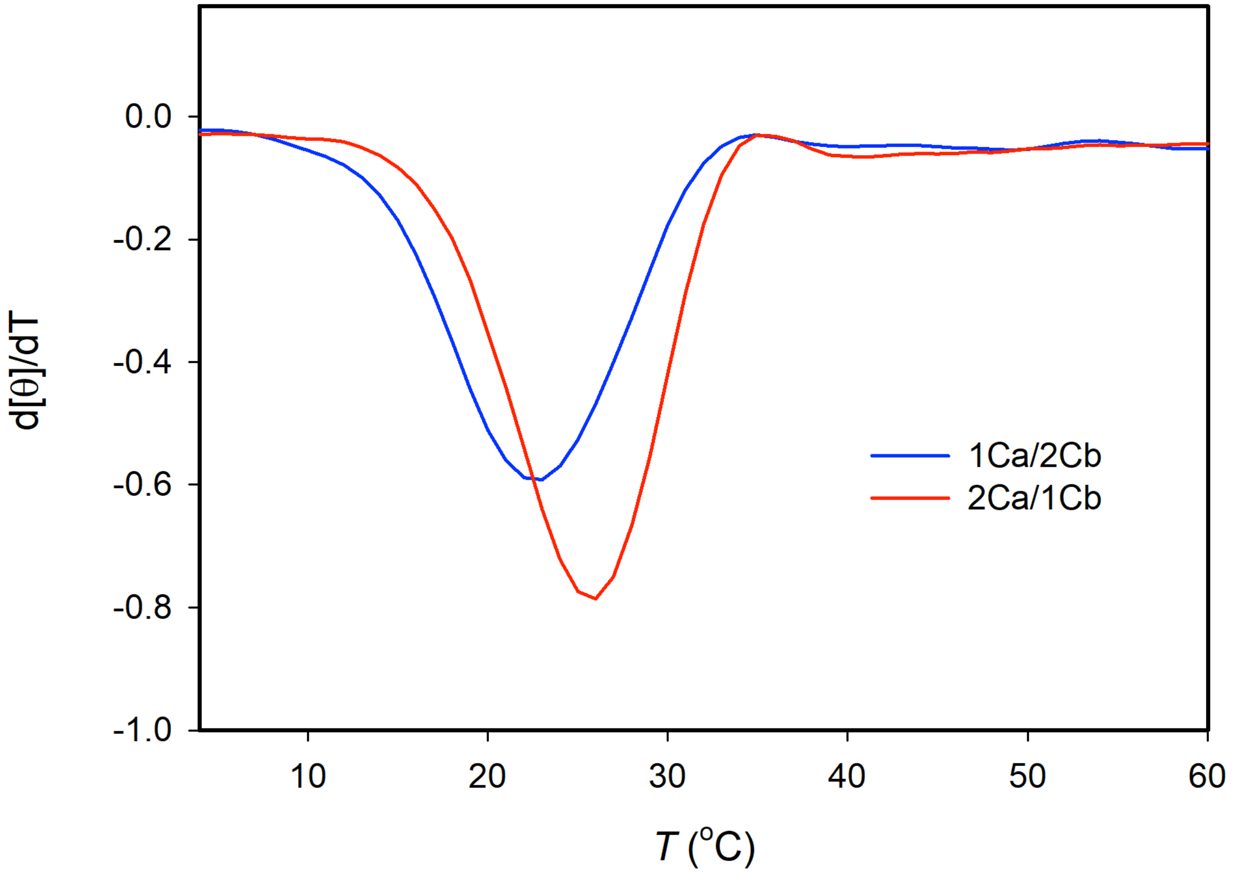

Figure S13. Plots of the first derivatives of molar ellipticity versus temperature for the thermal unfolding curves of 2Ca/1Cb and 1Ca/2Cb solutions. The T_m values are 26 °C for 2Ca/1Cb and 23 °C for 1Ca/2Cb.

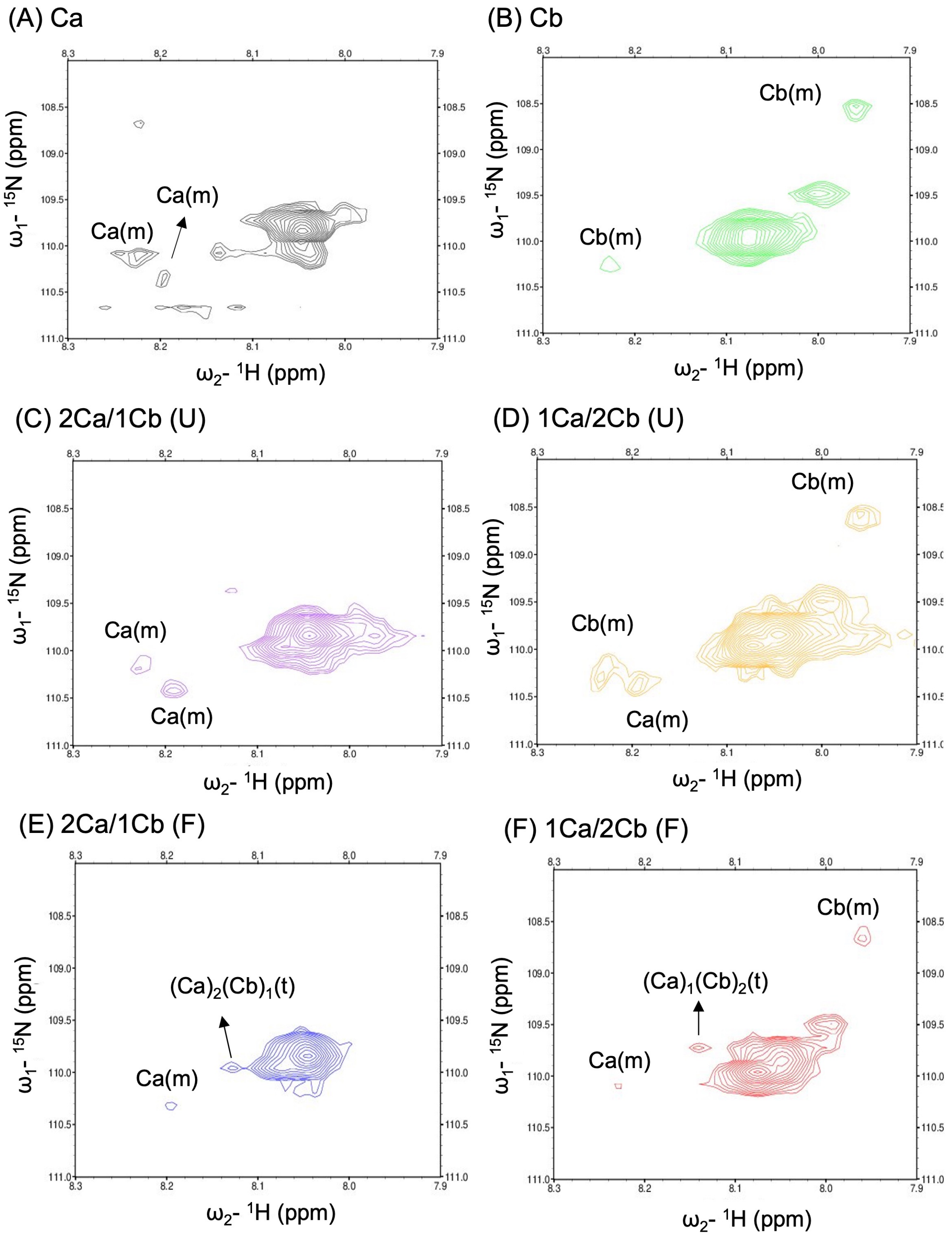

Figure S14. ^{1}H-^{15}N HSQC spectra for Ca, Cb, and their mixtures in different molar ratios. The (U) indicates the measurements were performed after heating the samples without incubation. The (F) indicates the measurements were conducted after the samples were incubated at 4 °C.

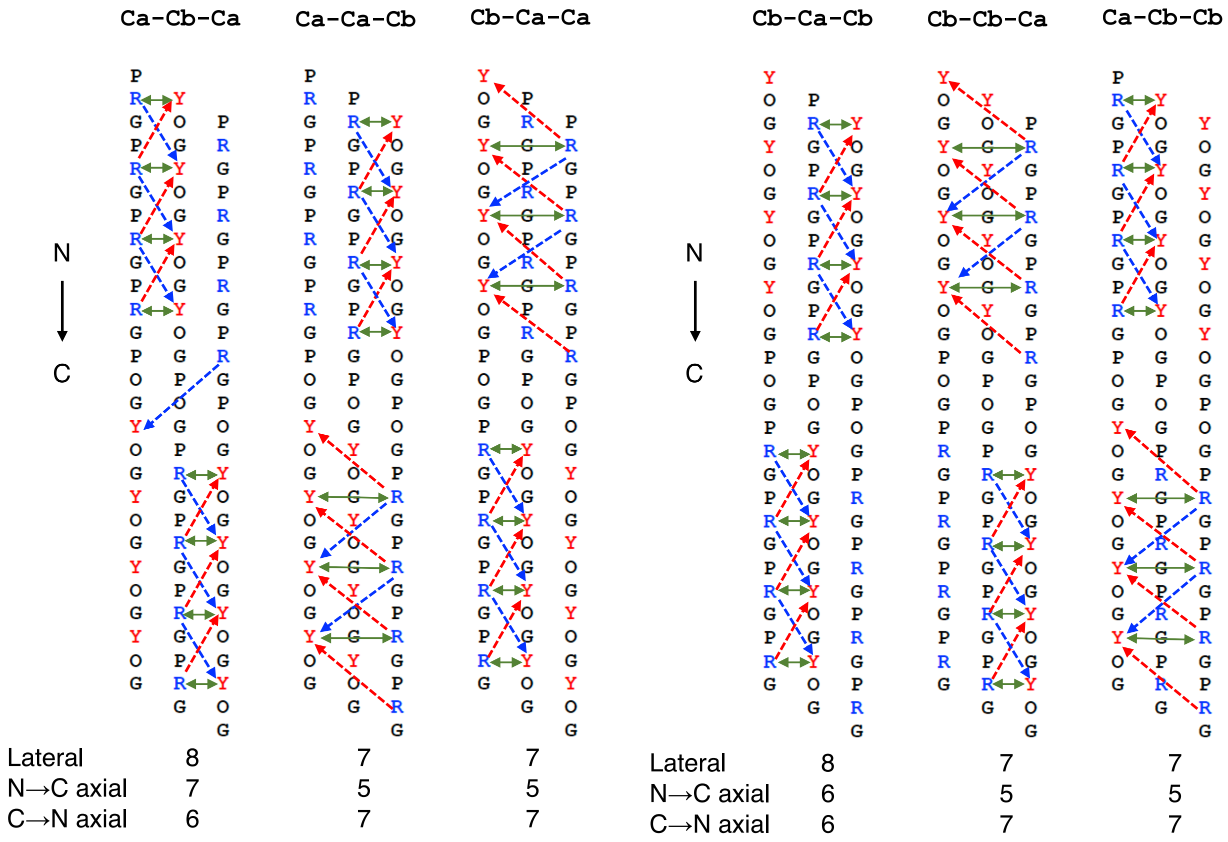

Figure S15. Different strand arrangements for the heterotrimers $(Ca)_2(Cb)_1$ and $(Ca)_1(Cb)_2$. The blue arrows indicate the N→C axial pairs, the red arrows indicate the C→N axial pairs, and the dark green arrows indicate the lateral pairs. The bottom panel shows the numbers of each type of pair.
